# Supplementary material for: Prior experience conditionally inhibits the expression of new learning in Drosophila
Source: Curr Biol. 2021 Aug 23;31(16):3490–3503.e3. doi: 10.1016/j.cub.2021.05.056 (PMC8409488; doi:10.1016/j.cub.2021.05.056)
Supplement: Document S1. Figures S1 and S2 [file mmc1.pdf]

Current Biology, Volume *31*

Supplemental Information

Prior experience conditionally inhibits the expression of new learning in *Drosophila*

Pedro F. Jacob, Paola Vargas-Gutierrez, Zeynep Okray, Stefania Vietti-Michelina, Johannes Felsenberg, and Scott Waddell

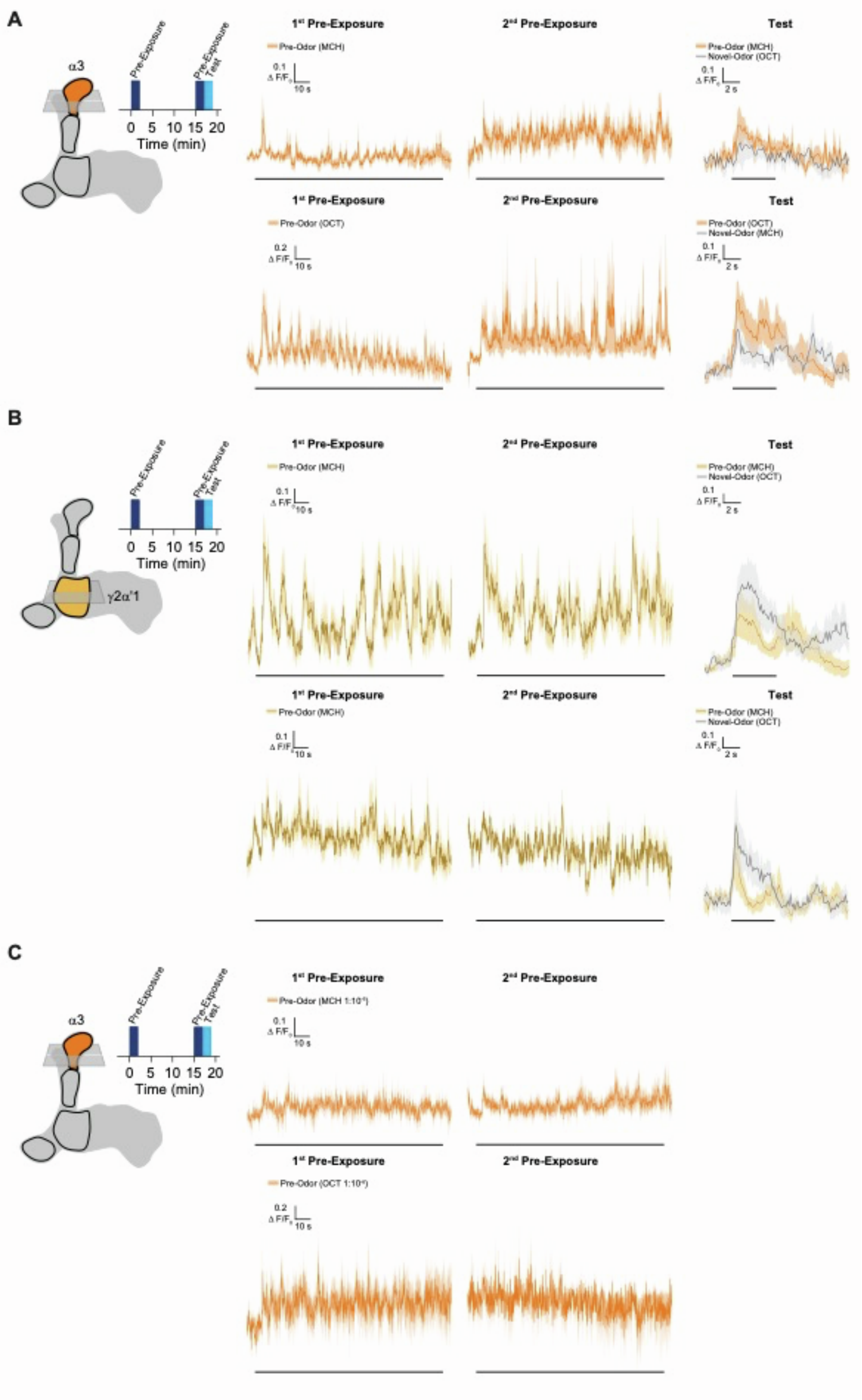

Figure S1. PPL1-α3 and PPL1-γ2α′1 DAN activity is altered following odor exposure. Related to Figure 4.

(**A**) Left: Imaging plane through the presynaptic field of an α3 DAN and training and imaging protocol. Middle: α3 DAN odor-evoked calcium responses during the 1st and 2nd pre-exposures to odor X (Pre-odor; either MCH or OCT, orange). Right: α3 DAN odor-evoked calcium

responses during the testing phase to odor X (Pre-odor, orange) and to odor Y (Novel-odor, gray). (**B**) Left: Imaging plane through the presynaptic field of a γ2α′1 DAN and training and imaging protocol. Middle: γ2α′1 DAN odor-evoked calcium responses during the 1st and 2nd pre-exposures to odor X (Pre-odor; either MCH or OCT, gold). Right: γ2α′1 DAN odor-evoked calcium responses during the testing phase to odor X (Pre-odor, gold) and to odor Y (Novel-odor, gray). (**C**) Left: Imaging plane through the presynaptic field of an α3 DAN and training and imaging protocol. Middle: α3 DAN odor-evoked calcium responses during 1st and 2nd pre-exposures to lower 10^{-6} concentration of odor X (Pre-odor; either 1:10^{-6} MCH or OCT, orange). In all panels, the horizontal line underneath the traces denotes the period of odor presentation. Odor-evoked activity traces show mean (solid line) with SEM (shadow). Data quantification in Figure 4.

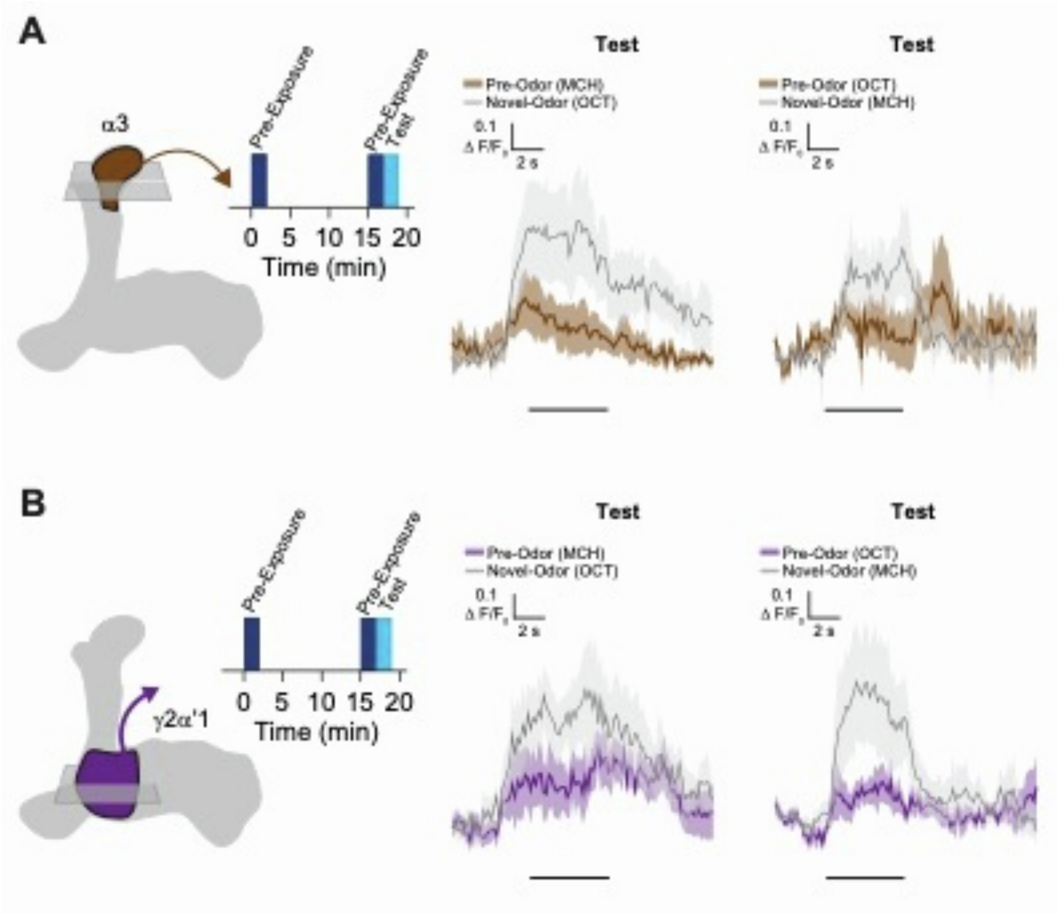

Figure S2. α3 and γ2α′1 MBONs exhibit an odor pre-exposure memory trace. Related to Figure 5.

(**A**) Left: Imaging plane through the dendritic field of an α3 MBON and training and imaging protocol. Right: α3 MBON odor-evoked calcium responses during the testing phase to odor X (Pre-odor, brown) and to odor Y (Novel-odor, gray). (**B**) Left: Imaging plane through the dendritic field of a γ2α′1 MBON and training and imaging protocol. Right: γ2α′1 MBON odor-evoked calcium responses during the testing phase to odor X (Pre-odor, purple) and to the odor Y (Novel-odor, gray). In all panels, the horizontal line underneath the traces denotes the period of odor presentation. Odor-evoked activity traces show mean (solid line) with SEM (shadow). Data quantification in Figure 5.
