# Supplementary material for: Prior experience conditionally inhibits the expression of new learning in Drosophila
Source: Curr Biol. 2021 Aug 23;31(16):3490–3503.e3. doi: 10.1016/j.cub.2021.05.056 (PMC8409488; doi:10.1016/j.cub.2021.05.056)
Supplement: Document S2. Article plus supplemental information [file mmc2.pdf]

Article

Current Biology

Prior experience conditionally inhibits the expression of new learning in *Drosophila*

Highlights

- Odor pre-exposure alters the expression of a learned association of that odor
- Pre-exposure memory only affects subsequent retrieval if context is consistent
- Pre-exposure memory can complement or compete with a learned association
- Odor pre-exposure forms a labile mushroom body-dependent aversive memory

Authors

Pedro F. Jacob,
Paola Vargas-Gutierrez, Zeynep Okray,
Stefania Vietti-Michelina,
Johannes Felsenberg, Scott Waddell

Correspondence

scott.waddell@cncb.ox.ac.uk

In brief

Jacob et al. show that prior experience of an odor can inhibit the retrieval of a newly formed memory for that same odor. Odor pre-exposure forms a labile mushroom body-dependent aversive memory that competes with expression of new learning if the context is appropriate. This study explains how a retrieval defect can underlie latent inhibition.

Jacob et al., 2021, Current Biology *31*, 3490–3503
August 23, 2021 © 2021 The Author(s). Published by Elsevier Inc.
https://doi.org/10.1016/j.cub.2021.05.056

CellPress

CellPress
OPEN ACCESS

Current Biology

Article

Prior experience conditionally inhibits the expression of new learning in *Drosophila*

Pedro F. Jacob,[1] Paola Vargas-Gutierrez,[1] Zeynep Okray,[1] Stefania Vietti-Michelina,[1,3] Johannes Felsenberg,[1,2] and Scott Waddell[1,4,5,*]
[1]Centre for Neural Circuits and Behaviour, University of Oxford, Oxford OX1 3TA, UK
[2]Present address: Friedrich Miescher Institute for Biomedical Research, Basel, Switzerland
[3]Present address: Department of Physiology, Anatomy and Genetics, University of Oxford, Parks Road, Oxford OX1 3SR, UK
[4]Twitter: @scottishwaddell
[5]Lead contact
*Correspondence: scott.waddell@cncb.ox.ac.uk
https://doi.org/10.1016/j.cub.2021.05.056

SUMMARY

Prior experience of a stimulus can inhibit subsequent acquisition or expression of a learned association of that stimulus. However, the neuronal manifestations of this learning effect, named latent inhibition (LI), are poorly understood. Here, we show that prior odor exposure can produce context-dependent LI of later appetitive olfactory memory performance in *Drosophila*. Odor pre-exposure forms a short-lived aversive memory whose lone expression lacks context-dependence. Acquisition of odor pre-exposure memory requires aversively reinforcing dopaminergic neurons that innervate two mushroom body compartments—one group of which exhibits increasing activity with successive odor experience. Odor-specific responses of the corresponding mushroom body output neurons are suppressed, and their output is necessary for expression of both pre-exposure memory and LI of appetitive memory. Therefore, odor pre-exposure attaches negative valence to the odor itself, and LI of appetitive memory results from a temporary and context-dependent retrieval deficit imposed by competition with the parallel short-lived aversive memory.

INTRODUCTION

Keeping track of life experience allows animals to benefit from all of their prior knowledge when learning new information and using their memory to direct behavior. Although the subject of great early debate among learning theorists, it is now accepted that learning occurs even without explicit rewards or punishment. Classic experiments showed that rats given the prior opportunity to roam in an empty maze performed better when they were later trained with rewards presented in specific locations.[1] Becoming familiar with the maze without explicit reinforcement and an obvious initial change in the animal's behavior was called "latent learning."[2]

Attempts to replicate a facilitating effect of latent learning using classical conditioning led to an unexpected observation. Pre-exposing animals to a stimulus instead often inhibited the ability of the animal to learn using that stimulus—a phenomenon given the name "latent inhibition" (LI).[3,4] LI has been heavily studied for the last 50 years, and two alternative theories have been proposed to account for the inhibitory effect of stimulus pre-exposure. In the acquisition (A) model, subsequent learning is considered to be impaired because pre-exposure alters the capacity for the stimulus to enter into new associations.[5–7] In contrast, in the retrieval (R) model, learning is still believed to occur but memory expression is impaired.[8–11] A strong argument in favor of the R model is the observation that LI often appears to be limited in time, leading to expression of the subsequent learning undergoing "spontaneous recovery." Importantly, both theories of latent inhibition assume that something is learned during pre-exposure such as primitive properties of the stimulus including its specific identity, intensity (e.g., concentration), and salience. In addition, LI is often sensitive to the consistency of the context within which the animal is pre-exposed, taught, and tested for memory expression. This led to the proposal that first learning an association between the stimulus and its context makes it difficult for the animal to subsequently associate the stimulus with reinforcement during training.[12,13]

Studying olfactory learning in the relatively small brain of *Drosophila* has potential to define how LI can operate and reveal an underlying neuronal circuit mechanism. Several earlier studies in both adult flies and larvae demonstrated that repeated exposure to an odor can alter its apparent valence to the fly, either making the fly avoid it more or become unresponsive to it.[14–18] Although an A model for LI has been reported with appetitive conditioning in the honeybee,[19–21] a prior study in adult *Drosophila* did not observe any effect on aversive conditioning following a single odor pre-exposure.[22]

Associative olfactory learning in *Drosophila* relies on the neuronal circuitry of the mushroom body (MB). Individual odors are represented as activity in sparse and largely non-overlapping subpopulations of the ∼4,000 intrinsic neurons called Kenyon cells (KCs). Positive or negative valence can be assigned to these odor representations by anatomically discrete dopaminergic neurons (DANs) which, via dopamine receptor-directed

 © 2021 The Author(s). Published by Elsevier Inc.
This is an open access article under the CC BY license (http://creativecommons.org/licenses/by/4.0/).

CellPress
OPEN ACCESS

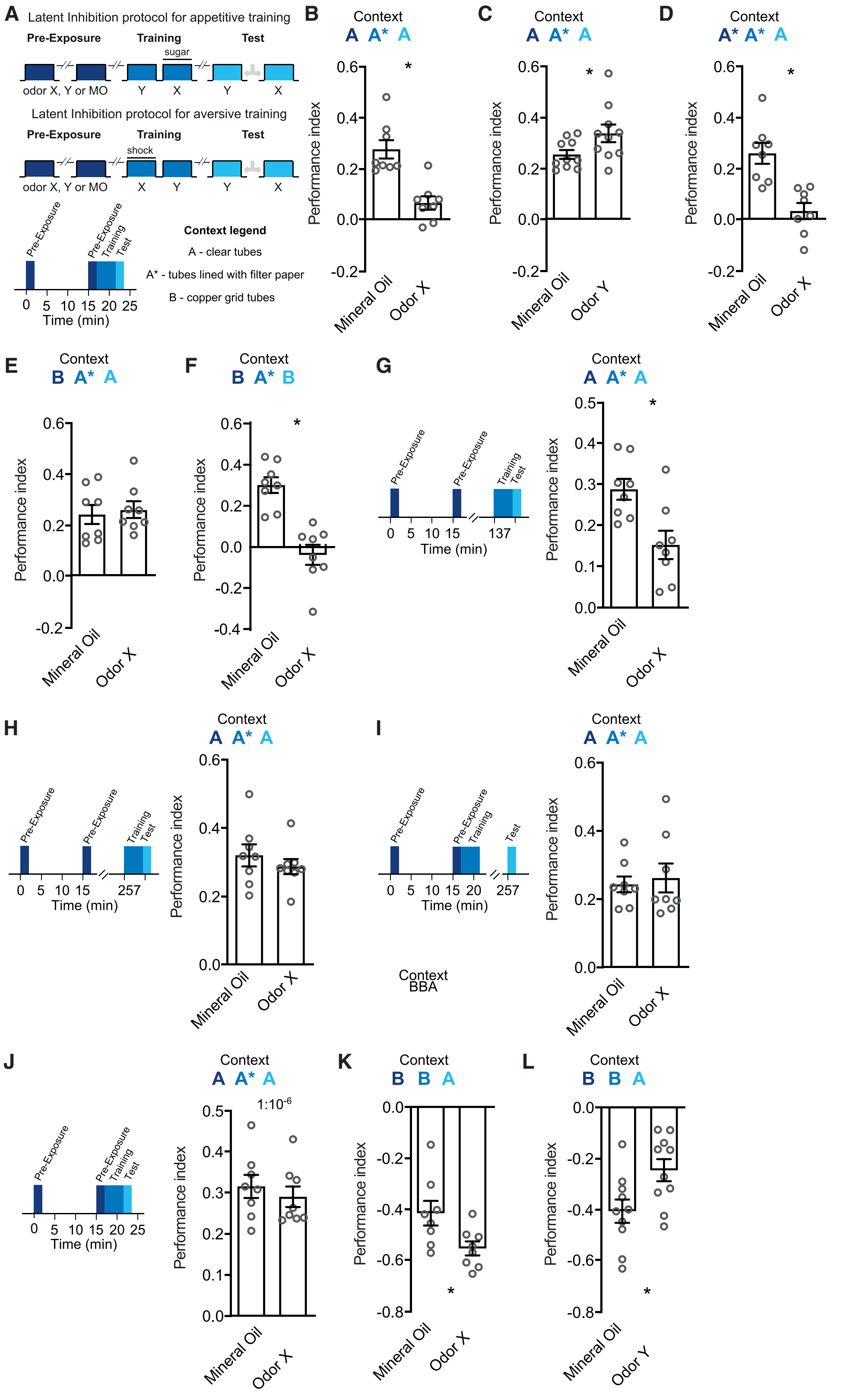

Figure 1. Odor pre-exposure induces context-dependent latent inhibition of appetitive learning

(A) Schematic for behavioral paradigms in this study. Flies were twice pre-exposed to either an odor X, Y, or mineral oil (MO), with a 15-min inter-trial interval (dark blue phase; pre-exposure). Flies were then either trained appetitively (with sugar) or aversively (with electric shock) (mid blue phase, training) and tested (light blue

(legend continued on next page)

cyclic AMP (cAMP)-dependent plasticity,[23–29] modulate the efficacy of KC output synapses onto different downstream mushroom body output neurons (MBONs), whose dendrites occupy the same MB compartment. Aversive learning depresses KC synapses onto MBONs whose activation favors approach, whereas appetitive learning reduces odor-drive to MBONs favoring avoidance. By establishing a skew in the valence of the odor-driven MBON network, learned information subsequently directs either odor avoidance or attraction behavior.[30]

A number of studies indicate that discrete experience is represented as plasticity of different combinations of KC-MBON connections, directed by the engagement of unique combinations of DANs. For example, different types of DANs have been implicated in coding memories for specific rewards (e.g., water, the sweet taste and nutrient value of sugars, the absence of expected shock, and the delayed recognition of safety).[31–36] In contrast, the same PPL1 DANs appear to be required to code aversive memories for electric shock, bitter taste, and heat,[37–39] although imaging suggests they are activated by temperature decreases and to noxious heat.[39,40] By forming and storing conflicting and complementary memories in different places, the fly can more effectively direct its behavior to reflect a history of experience.

Here, we show that prior odor exposure can temporarily inhibit memory performance after subsequent appetitive learning in *Drosophila*. This inhibitory effect is sensitive to a change of context across the pre-exposure, training, and testing periods, consistent with it being a form of LI. Odor pre-exposure forms a short-lived odor-specific aversive memory, whose acquisition requires the $\gamma 2\alpha' 1$ and $\alpha 3$ DANs, the latter of which become sensitized to consecutive odor presentation. As a consequence, aversive memory is apparent as a decrease in the odor-evoked response of the corresponding approach-directing $\gamma 2\alpha' 1$ and $\alpha 3$ MBONs. Blocking the $\alpha 3$ MBONs impairs the expression of the aversive odor pre-exposure memory and abolishes LI of appetitive memory. The short-lived presence of a parallel and differently located odor-specific aversive memory therefore temporarily inhibits the retrieval of a subsequently formed appetitive memory for that same odor. These data provide evidence for a context-dependent R model of latent inhibition in *Drosophila*.

RESULTS

Odor pre-exposure can produce temporary latent inhibition of appetitive memory

Drosophila can be appetitively conditioned by pairing odor presentation with sugar reward.[15,41] We therefore tested whether prior exposure to the to be sugar-paired odor (CS+) altered subsequent learned behavior. Starved flies were given two 2-min odor X presentations with a 15-min inter-trial interval (ITI) before being trained by presenting odor Y for 2 min then 30 s later presenting odor X with sucrose reward (two odors were reciprocally used as X and Y, and performance indices represent an average). Control flies were twice pre-exposed to the odor diluent of mineral oil (MO) before training (Figure 1A). When immediately tested after training, MO-exposed flies exhibited appetitive memory for odor X. However, the performance of flies pre-exposed to the conditioned odor X was significantly impaired (Figure 1B), a phenotype resembling LI. In contrast, flies pre-exposed to odor Y (the to be CS−) exhibited appetitive memory performance that was significantly enhanced to that of flies exposed to MO (Figure 1C). Because LI can typically be reduced by changing the context between pre-exposure, training, and testing, we next altered the

phase, test) for their preference between odors X and Y. To evaluate the effect of context, three different contexts were used: clear tubes (context A), tubes lines with filter paper (context A*), and copper grid tubes (context B). In subsequent figures, the changes in context between the pre-exposure, training, and test phases are marked using this nomenclature and are color-coded accordingly. For example, dark blue A, mid blue A*, and light blue A corresponds to clear tubes in the pre-exposure and test phases and tubes lined with filter paper in the training phase.

(B) Flies were twice pre-exposed to odor X or MO in clear tubes (context A), with a 15-min inter-trial interval. Immediately following the last exposure, they were trained by presenting odor Y for 2 min, then odor X for 2 min with sugar (CS+) in context A*. Flies pre-exposed to odor X exhibited appetitive memory performance to odor X (one-sample t test: t(7) = 2.419, p = 0.0461) that was significantly reduced (LI) in comparison to flies exposed to MO (t(14) = 4.719, p = 0.0003).

(C) When pre-exposed to odor Y (the to be CS−; context A), flies exhibited appetitive memory that was significantly increased in comparison to flies exposed to MO (t(18) = 2.127, p = 0.0475).

(D) When pre-exposure and training context were more closely matched, using filter paper in the pre-exposure tube (context A*A*A), flies pre-exposed to odor X did not exhibit appetitive memory performance (LI) (one-sample t test: t(7) = 0.9866, p = 0.3567). Performance was also significantly reduced compared to flies pre-exposed to MO (t(14) = 4.376, p = 0.0006).

(E) Pre-exposing flies to odor X in a different context of a copper grid tube (context BA*A) abolished the inhibitory effect (no LI). Appetitive memory performance of flies pre-exposed to odor X was similar to MO pre-exposed flies (t(14) = 0.3902, p = 0.7023).

(F) Matching context between pre-exposure and testing (both in copper grid tube; context BA*B) restored LI. Performance of flies pre-exposed to odor X was significantly impaired compared to flies pre-exposed to MO (t(14) = 5.449, p < 0.0001).

(G) The inhibitory effect on appetitive learning is evident 2 h after odor pre-exposure. Flies were pre-exposed to odor X or MO. 2 h later, they were appetitively trained and tested immediately for memory. Performance of flies pre-exposed to odor X was significantly reduced compared to flies pre-exposed to MO (t(14) = 3.155, p = 0.007).

(H) The inhibitory effect on learning was not evident 4 h after odor pre-exposure. Memory performance of flies pre-exposed to odor X or MO was similar when trained 4 h after the last pre-exposure (t(14) = 0.8368, p = 0.4167).

(I) The inhibitory effect of pre-exposure on learning was not evident 4 h after training. Performance was similar if flies were pre-exposed to odor X or MO, then immediately trained but tested for memory 4 h later (t(14) = 0.7041), demonstrating time-dependent loss of LI and recovery of appetitive memory.

(J) Pre-exposure to lower odor concentration did not induce LI. Flies were pre-exposed with $1{:}10^{-6}$ odor X or mineral oil (MO), with 15-min inter-trial interval, in clear tubes, immediately trained with $1{:}10^{-6}$ odor X paired with sugar (CS+) and tested for memory. Flies pre-exposed to $1{:}10^{-6}$ odor X showed similar memory performance to those pre-exposed to MO (t(14) = 0.6741, p = 0.5112).

(K) Odor pre-exposure facilitates memory performance after aversive conditioning. Flies were pre-exposed to odor X or mineral oil (MO) in a copper grid-lined tube (context B), then immediately trained by pairing odor X for 1 min with twelve 30 V electric shocks (CS+). Flies pre-exposed to odor X exhibited increased aversive memory performance in comparison to MO-exposed flies (t(14) = 2.485, p = 0.0262).

(L) When pre-exposed to odor Y (the to be CS; context B), flies exhibited aversive memory that was significantly decreased in comparison to flies exposed to MO (t(18) = 2.560, p = 0.0197).

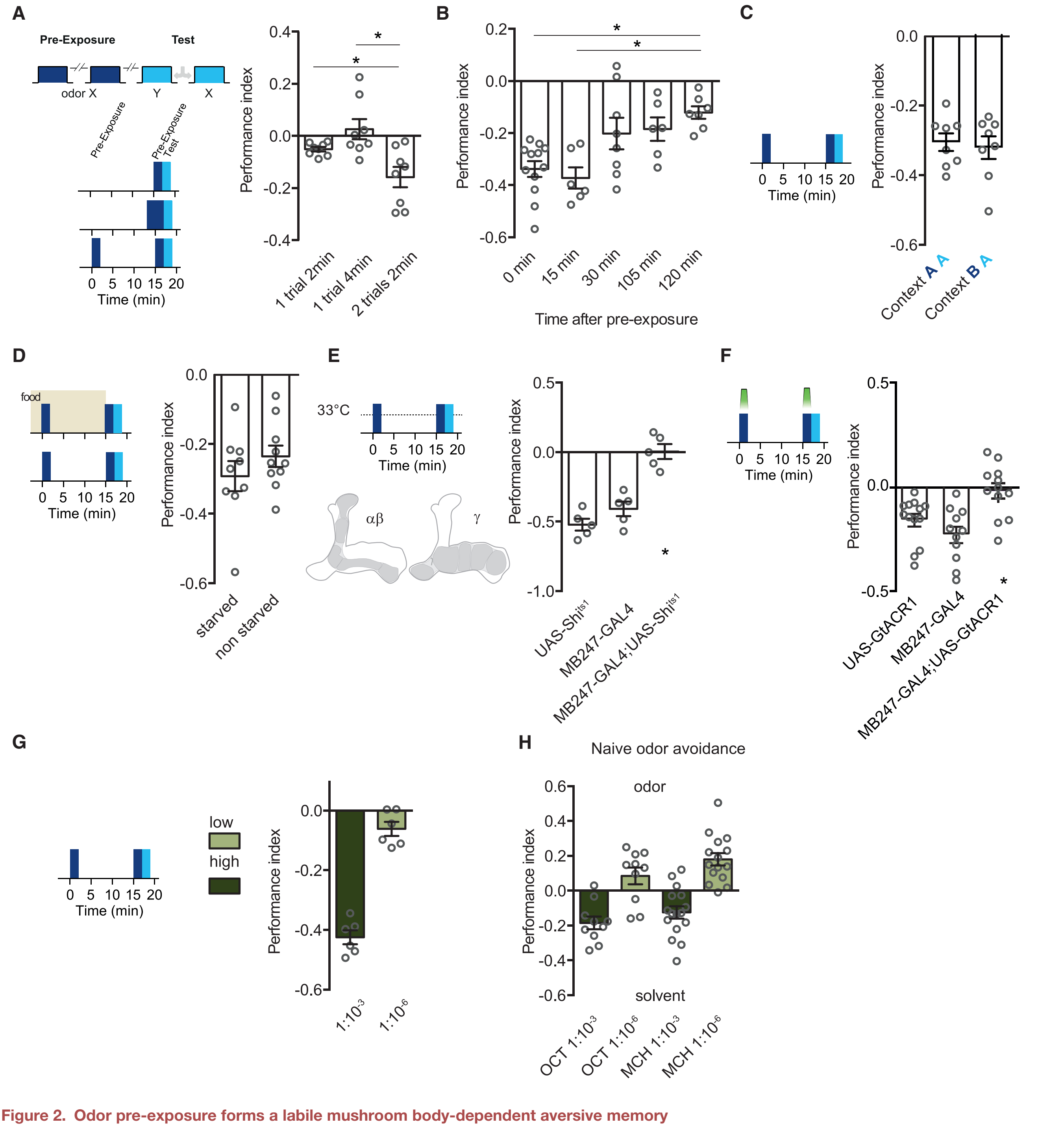

Figure 2. Odor pre-exposure forms a labile mushroom body-dependent aversive memory

(A) Repeated odor exposure forms aversive memory for that odor. Two 2-min odor exposures with a 15-min interval induced odor aversion ($F[2,21] = 8.365$, $p = 0.0023$, $n = 8$) whereas a single 2-min or 4-min exposure did not (Tukey's multiple comparisons test: 1 × 2 min versus 1 × 4 min, $p = 0.07$; 1 × 2 min versus 2 × 2 min, $p = 0.0182$; 1 × 4 min versus 2 × 2 min, $p = 0.0048$).

(B) Pre-exposure induced aversive memory is labile (one-sample t test $p < 0.05$ for all comparisons; $F[4,34] = 6.295$, $p = 0.007$, $n = 6–12$; Tukey's multiple comparisons test: 0 min versus 120 min, $p = 0.0036$ and 15 min versus 120 min, $p = 0.0040$, for all other comparisons $p > 0.05$).

(C) Expression of pre-exposure memory is not sensitive to changing context. Performance of flies pre-exposed in copper-lined and tested in clear tubes (context BA) was indistinguishable from that of flies pre-exposed and tested in clear tubes (context AA) ($t(14) = 0.3827$, $p = 0.7077$).

(D) Formation of odor pre-exposure memory does not depend on hunger ($t(17) = 1.090$, $p = 0.2911$).

(E) Blocking synaptic output from the MB KCs abolishes odor pre-exposure memory ($F[2,12] = 30.87$, $p < 0.0001$, $n = 5$; Tukey's multiple comparisons test: UAS-*Shi*^{ts1} versus MB247-GAL4, $p = 0.2773$; UAS-*Shi*^{ts1} versus MB247-GAL4;UAS-*Shi*^{ts1}, $p < 0.0001$; MB247-GAL4 versus MB247-GAL4;UAS-*Shi*^{ts1}, $p = 0.0002$).

(legend continued on next page)

context between these experimental stages. In our initial experiments (Figures 1B and 1C), the flies were pre-exposed in clear tubes, trained in tubes lined with filter paper, and then tested in clear tubes. We therefore pre-exposed and trained the flies in tubes lined with filter paper to make the context consistent during these stages. This constancy of pre-exposure and training context produced a particularly strong inhibitory effect of pre-exposure (Figure 1D). More importantly, these first two experiments suggest that flies may consider the context of clear (A) and paper (A*) tubes to be similar. We therefore next explicitly changed the context by pre-exposing flies to the to be CS+ in a copper grid-lined tube (B) before training them in tubes lined with filter paper (A*), and testing them in clear tubes (A) (Figure 1E). Strikingly, the robust change in context between copper grid tubes and paper/clear (BA*A) abolished the inhibitory effect of odor pre-exposure. A failure to observe LI across changing context could result from the inability of the flies to form or retrieve a pre-exposure memory. However, robust LI was recovered when the flies were trained on paper but were pre-exposed and tested in copper grid tubes (BA*B) (Figure 1F), showing that pre-exposure memory is formed but not retrieved in the BA*A scenario. Together, these context-shifting experiments demonstrate that the inhibitory effect of odor pre-exposure on subsequent appetitive memory is a form of LI whose expression depends on the context at testing approximating that experienced during odor pre-exposure. These experiments also suggest that this form of LI results from a retrieval deficit—an R model.

We next tested the persistence of LI. Whereas pre-exposure to the CS+ impaired memory performance when flies were trained 2 h after the last pre-exposure (Figure 1G), performance was unimpaired if training was delayed for 4 h after the last pre-exposure (Figure 1H). In addition, appetitive memory performance was unimpaired if flies were trained immediately after the second pre-exposure but tested 4 h later (Figure 1I). These experiments, therefore, suggest that the inhibitory effect of LI decays between 2 and 4 h after odor pre-exposure. In addition, the recovery of appetitive memory performance is also consistent with LI resulting from a temporary retrieval deficit, which provides further evidence that an R model is in operation here.

Many studies have shown that learning and LI is sensitive to properties (frequency, amount, and duration) of the conditioned stimulus.[42–44] We therefore also tested whether two exposures of a lower odor concentration (10^{-6} rather than 10^{-3}) with 15 min ITI induced LI of appetitive memory. Although training with this odor concentration produced robust appetitive memory, no LI effect was observed following odor pre-exposure (Figure 1J).

Odor pre-exposure can produce facilitation of aversive memory

We also tested whether odor pre-exposure altered performance measured after aversive training, pairing odor with electric shock. Starved flies were given two 2-min odor X presentations with a 15 min ITI before being trained by presenting odor X for 1 min, paired with electric shocks, then 45 s later presenting odor Y (Figure 1A). Surprisingly, flies pre-exposed to the CS+ exhibited enhanced aversive memory performance as compared to flies pre-exposed to MO (Figure 1K). This enhancement may represent a form of "latent faciliation."[45,46] Finding that the same odor pre-exposure schedule can inhibit appetitive memory but facilitate aversive memory performance led us to hypothesize that pre-exposure might form an avoidance memory for the CS+; in the former case, this is competing, and in the latter case, this is complementary. In further support of this notion, flies pre-exposed to odor Y (the to be CS−) exhibited aversive memory performance that was significantly impaired to that of MO pre-exposed flies (Figure 1L)—a prediction of the flies acquiring an aversive pre-exposure memory for the CS− and a shock-reinforced aversive memory for the CS+. Importantly, finding that CS+ pre-exposure does not inhibit aversive learning argues against an A model accounting for the observed LI, because both appetitive and aversive learning should be reduced if pre-exposure prevented the CS+ entering into a learned association.

Pre-exposure forms a short-lived MB-dependent aversive odor memory

Although repeated odor presentation frequently leads to rapid sensory adaptation of insect olfactory sensory neurons and/or behavioral habituation, these effects often require many trials, and the response decrement decays within a few minutes.[17,18,47–51] Prior work has also shown that a few odor pre-exposures can enhance subsequent odor avoidance behavior in *Drosophila*,[14,15,34] consistent with our hypothesized aversive learning model. We therefore tested whether our LI odor exposure regimen altered odor avoidance behavior. As in the prior experiments, starved flies were exposed twice to 2 min of odor X (10^{-3} dilution in MO) with a 15 min ITI. They were then immediately tested for preference between the pre-exposed odor X and another odor Y without any training. Flies pre-exposed twice to odor X showed a selective avoidance of odor X, consistent with pre-exposure forming an aversive odor X memory (Figure 2A). In contrast, a single odor pre-exposure, of either 2 or 4 min, did not alter odor preference, suggesting that repeated exposure is required to form the avoidance memory (Figure 2A). Measuring odor preference at different times after pre-exposure revealed that the avoidance memory is labile and slowly decays between 15 min and 2 h (Figure 2B). We also tested whether expression of pre-exposure memory was sensitive to context by pre-exposing flies to odor in copper grid tubes and testing them for odor preference in clear tubes. Performance was unaffected by this change of context (Figure 2C), demonstrating that context is uniquely important for the LI effect of pre-exposure memory. We last tested whether the explicit absence of food acts as

(F) Forming pre-exposure memory requires KC activity during odor pre-exposure. Restricting KC block to the period of odor pre-exposure, using GtACR1 and green light, abolishes aversion to odor X ($F[2,33] = 9.088$, $p = 0.0007$, $n = 11–13$; Tukey's multiple comparisons test: UAS-*GtACR1* versus MB247-GAL4, $p = 0.3423$; UAS-*GtACR1* versus MB247-GAL4;UAS-*GtACR1*, $p = 0.0183$; MB247-GAL4 versus MB247-GAL4;UAS-*GtACR1*, $p = 0.0006$).

(G) Formation of pre-exposure memory depends on odor concentration. Pre-exposure to high (10^{-3}) but not low (10^{-6}) concentration of odor induces aversive odor memory ($t(10) = 10.98$, $p < 0.0001$).

(H) Reducing odor concentration by 3 orders of magnitude can switch naive valence from avoidance to attraction. All pre-exposure experiments were performed in the context A (clear tubes) for pre-exposure and testing phases.

CellPress
OPEN ACCESS

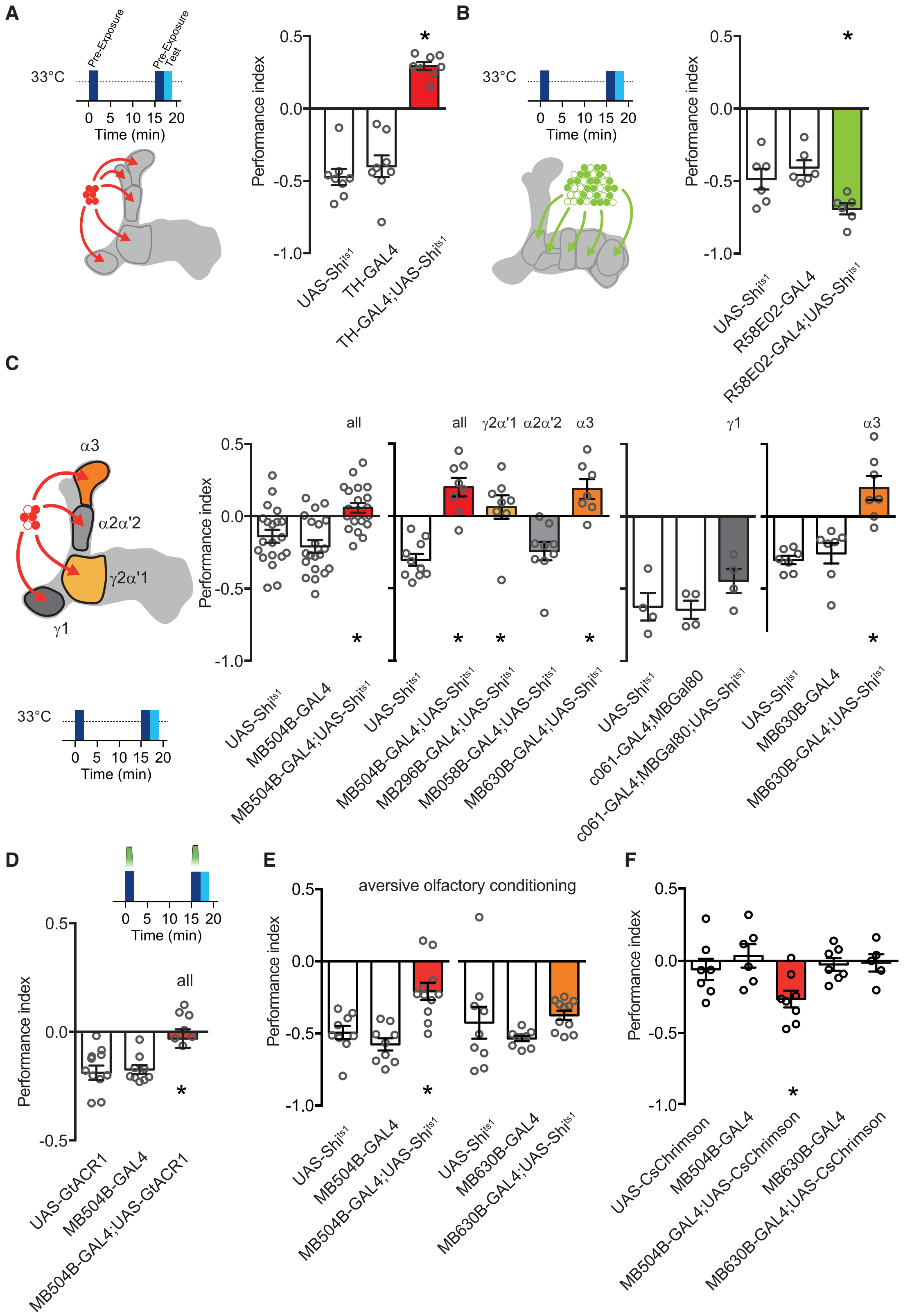

(legend on next page)

aversive reinforcement for hungry flies by pre-exposing satiated flies. However, pre-exposure induced similar odor avoidance in satiated flies as compared to starved flies (Figure 2D). Because our LI effect was assayed in hungry flies, all subsequent experiments in this study were performed in hungry flies.

Olfactory memories typically depend on the neuronal circuitry of the MB.[41,52–56] We therefore tested the consequence of blocking output from αβ and γ subsets of MB KCs on the effect of odor pre-exposure. We expressed the dominant temperature-sensitive UAS-*Shibire*^{ts1} (UAS-*Shi*^{ts1}) transgene[57] with MB247-GAL4 and blocked KC output throughout the experiment by raising the temperature to restrictive 33°C. This manipulation abolished the development of odor avoidance performance (Figure 2E). Moreover, restricting inhibition of KC activity to only the periods of odor pre-exposure using expression of the green light-sensitive anion-selective GtACR1 channel[58] revealed that KC activity is necessary during pre-exposure (Figure 2F); this suggests that it is a MB-dependent process.

Because we observed LI when flies were twice pre-exposed to 10^{-3} odor concentration but not to 10^{-6}, we also tested whether this lower odor concentration produced an aversive pre-exposure memory. Consistent with the lack of LI, no enhanced avoidance was observed following 10^{-6} odor exposures (Figure 2G). In addition, when naive flies were given the choice between a 10^{-3} odor stream and air, they exhibited avoidance of the odor (Figure 2H). In contrast, flies either showed no preference, or odor approach when tested with a 10^{-6} odor stream and air. Therefore, we reasoned that lower odor concentrations, which are less repellent,[59] do not act as an aversive reinforcer during odor pre-exposure.

Pre-exposure memory requires γ2α′1 and α3 DANs

Aversive olfactory learning reinforced by electric shock, bitter taste, or heat depends on punishment coding DANs from the PPL1 cluster.[37,38,60–63] In contrast, DANs in the PAM cluster mostly code for reward.[31,35,64] We therefore first used TH-GAL4 and R58E02-GAL4 to express UAS-*Shi*^{ts1} and test the respective roles of PPL1 and PAM DANs in pre-exposure learning. Blocking TH-GAL4 neurons switched the effect of pre-exposure from generating aversion to approach (Figure 3A). This reversal of valence implies that removing aversive signaling may either release (mutually exclusive) or unmask (in parallel) positive reinforcement induced by odor pre-exposure. Blocking the rewarding DANs with R58E02-GAL4; UAS-*Shi*^{ts1} increased the aversive effect of pre-exposure (Figure 3B), consistent with odor exposure engaging negative and positive reinforcing DAN populations in parallel.

More specifically, blocking the four types of PPL1-DANs implicated in shock-reinforced olfactory learning,[63] either throughout the experiment (Figure 3C) or only during odor pre-exposure (Figure 3D), abolished the learned aversion. Blocking the individual types revealed that the PPL1-γ1pedc (MB-MP1) and PPL1-α′2α2 DANs are dispensable for pre-exposure learning. In contrast, blocking PPL1-γ2α′1 DANs (MB-MV1) abolished pre-exposure learning, whereas blocking the PPL1-α3 DANs converted pre-exposure-induced aversion to approach (Figure 3C).

Because PPL1-DANs are thought to provide an aversive teaching signal, common to electric shock, heat, and bitter taste,[37,38] we also tested for a role of PPL1-α3 DANs in shock-reinforced aversive memory. Although blocking the four PPL1-DANs impaired shock learning, blocking only the PPL1-α3 DANs left immediate shock memory intact (Figure 3E). In addition, replacing shock with optogenetic stimulation of the four PPL1-DANs could artificially implant an aversive memory, whereas a single pairing of odor presentation with PPL1-α3 DAN activation did not form aversive memory (Figure 3F). These data are consistent with a prior study that showed learning requires multiple trials of PPL1-α3 DAN activation.[65] These results demonstrate that two-trial pre-exposure learning and single-trial electric shock learning involve different PPL1-DANs and emphasize the importance of PPL1-α3 DANs for pre-exposure learning.

Figure 3. Forming pre-exposure memory requires γ2α′1 and α3 dopaminergic neurons

(A) Blocking punishment coding DANs switches the valence of odor pre-exposure learning. Exposure induced aversion becomes approach ($F[2,21] = 57.26$, $p < 0.0001$, n = 8; Tukey's multiple comparisons test: UAS-*Shi*^{ts1} versus TH-GAL4, p = 0.6528;UAS-*Shi*^{ts1} versus TH-GAL4;UAS-*Shi*^{ts1}, $p < 0.0001$; TH-GAL4 versus TH-GAL4; UAS-*Shi*^{ts1}, $p < 0.0001$).

(B) Blocking reward coding DANs increases the aversive effect of odor pre-exposure ($F[2,15] = 7.126$, p = 0.0067, n = 6; Tukey's multiple comparisons test: UAS-*Shi*^{ts1} versus R58E02-GAL4, p = 0.5676; UAS-*Shi*^{ts1} versus R58E02-GAL4/UAS-*Shi*^{ts1}, p = 0.0473; R58E02-GAL4 versus R58E02-GAL4/UAS-*Shi*^{ts1}, p = 0.0062).

(C) Pre-exposure-evoked aversive learning requires PPL1 DANs ($F[2,57] = 11.30$, $p < 0.0001$, n = 20; Tukey's multiple comparisons test: UAS-*Shi*^{ts1} versus MB504B-GAL4, p = 0.4573; UAS-*Shi*^{ts1} versus MB504B-GAL4;UAS-*Shi*^{ts1}, p = 0.0037; MB504B-GAL4 versus MB504B-GAL4;UAS-*Shi*^{ts1}, $p < 0.0001$). A specific requirement was observed for PPL1-γ2α′1 (MV1) and PPL1-α3 DANs (for PPL1 screen: $F[4,37] = 14.80$, $p < 0.0001$, n = 7–10; Dunnett's multiple comparisons test: UAS-*Shi*^{ts1} versus MB504B-GAL4;UAS-*Shi*^{ts1}, $p < 0.0001$; UAS-*Shi*^{ts1} versus MB296B-GAL4;UAS-*Shi*^{ts1}, p = 0.0006; UAS-*Shi*^{ts1} versus MB058B-GAL4;UAS-*Shi*^{ts1}, p = 0.8902; UAS-*Shi*^{ts1} versus MB630B-GAL4;UAS-*Shi*^{ts1}, $p < 0.0001$; for PPL1-γ1: $F[2,9] = 1.785$, p = 0.2223, n = 4; for PPL1-α3: $F[2,29] = 23.16$, $p < 0.0001$, n = 10–11; Tukey's multiple comparisons test: UAS-*Shi*^{ts1} versus MB630B-GAL4, p = 0.5575; UAS-*Shi*^{ts1} versus MB630B-GAL4;UAS-*Shi*^{ts1}, $p < 0.0001$; MB630B-GAL4 versus MB630B-GAL4;UAS-*Shi*^{ts1}, $p < 0.0001$).

(D) Optogenetic silencing of PPL1 DANs during odor exposures blocks exposure-evoked aversive learning ($F[2,24] = 6.325$, p = 0.0062, n = 9; Tukey's multiple comparisons test: UAS-*GtACR1* versus MB504B-GAL4, p = 0.9832;UAS-*GtACR1* versus MB504B-GAL4;UAS-*GtACR1*, p = 0.0112; MB504B-GAL4 versus MB504B-GAL4;UAS-*GtACR1*, p = 0.0169).

(E) Blocking PPL1 DANs impairs immediate aversive memory following conditioning pairing odor with 90V electric shocks ($F[2,26] = 14.43$, $p < 0.0001$, n = 9–11; Tukey's multiple comparisons test: UAS-*Shi*^{ts1} versus MB504B-GAL4, p = 0.5452; UAS-*Shi*^{ts1} versus MB504B-GAL4;UAS-*Shi*^{ts1}, p = 0.0016; MB504B-GAL4 versus MB504B-GAL4;UAS-*Shi*^{ts1}, $p < 0.0001$). However, only blocking the PPL1-α3 DANs has no effect ($F[2,26] = 1.692$, p = 0.2039, n = 9–11).

(F) Pairing odor exposure with optogenetic activation of PPL1 DANs induces aversive memory to the previously paired odor, whereas activation of only PPL1-α3 DANs is ineffective ($F[4,41] = 5.665$, p = 0.0010, n = 7–11; Bonferroni's multiple comparisons test: UAS-CsChrimson versus MB504B-GAL4, $p > 0.9999$; UAS-CsChrimson versus MB504B-GAL4;UAS-CsChrimson, p = 0.0025; MB504B-GAL4 versus MB504B-GAL4;UAS-CsChrimson, p = 0.0028; UAS-CsChrimson versus MB630B-GAL4, $p > 0.9999$; UAS-CsChrimson versus MB630B-GAL4;UAS-CsChrimson, $p > 0.9999$; MB630B-GAL4 versus MB504B-GAL4;UAS-CsChrimson, $p > 0.9999$). All pre-exposure experiments were performed in the context A (clear tubes) for pre-exposure and testing phases.

CellPress
OPEN ACCESS

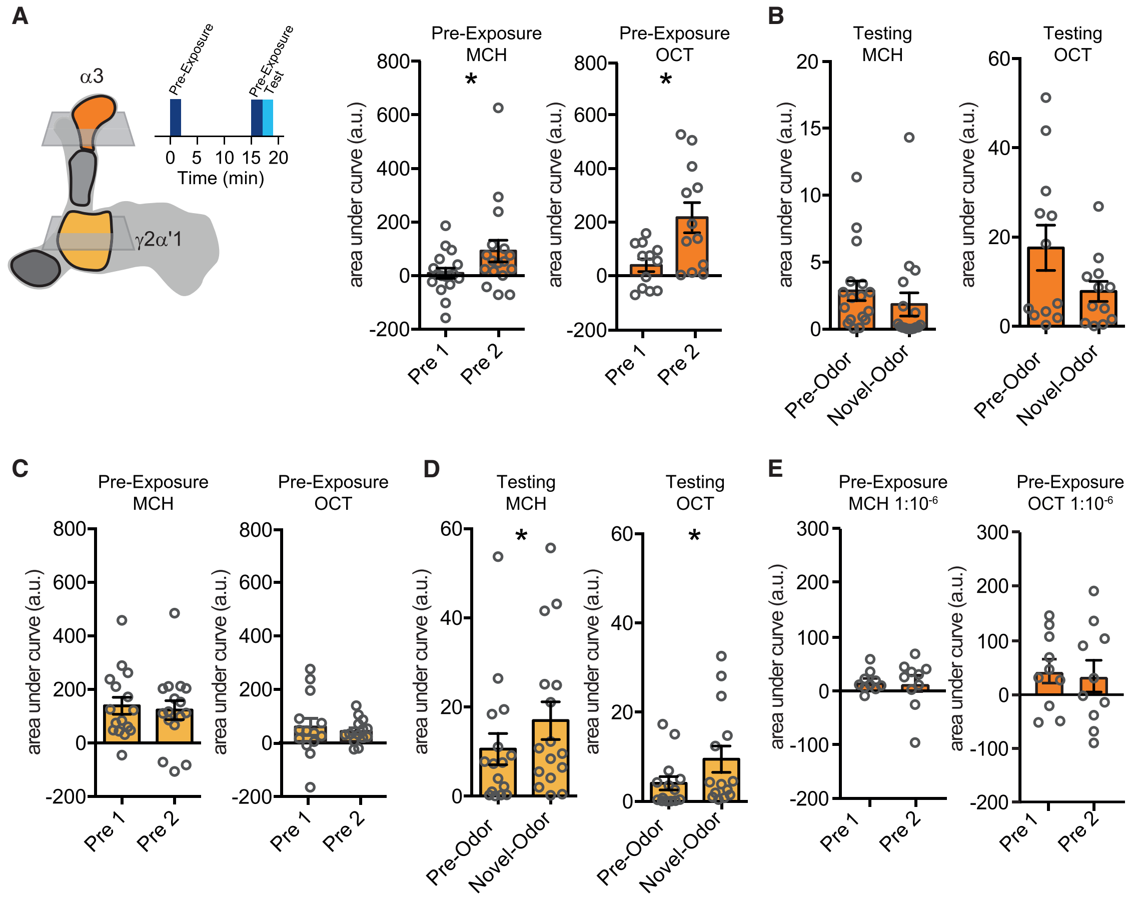

Figure 4. PPL1-γ2α′1 and PPL1-α3 DAN activity is altered following odor exposure

(A) Odor-evoked responses of PPL1-α3 DANs increase from the first to the second odor presentation (for MCH: $W(18) = 91$, $p = 0.0438$; for OCT: $t(11) = 2.859$, $p = 0.0156$).

(B) In the test responses of PPL1-α3 DANs to the pre-exposed odor were not different to those for the novel odor (for MCH: $W(18) = -33$, $p = 0.4951$; for OCT: $W(12) = -40$, $p = 0.1294$).

(C) Odor-evoked responses of PPL1-γ2α′1 DANs did not change between the first and second odor presentations (for MCH: $t(17) = 0.4792$, $p = 0.6379$; for OCT: $t(13) = 0.5678$, $p = 0.5798$).

(D) In the test, responses of PPL1-γ2α′1 DANs to the pre-exposed odor were reduced compared to those for the novel odor (for MCH: $W(18) = 107$, $p = 0.0182$; for OCT: $W(14) = 71$, $p = 0.0245$).

(E) Responses of PPL1-α3 DANs did not change when flies were twice exposed to lower concentration of odor (for MCH: $t(9) = 0.1930$, $p = 0.8513$; for OCT: $t(9) = 0.2901$, $p = 0.7783$). Imaging traces are presented in Figure S1.

The activity of PPL1-γ2α′1 and PPL1-α3 DANs is altered following odor presentation

Because pre-exposure learning depends on repeated trials and requires the PPL1-γ2α′1 and PPL1-α3 DANs, we imaged DAN activity during and after odor presentations under the microscope (Figures 4 and S1). Flies were constructed that expressed the fluorescent UAS-GCaMP6m calcium sensor in PPL1-γ2α′1 or PPL1-α3 DANs. As before in the behavioral experiments, flies were given two odor presentations with 15 min ITI. They were then immediately given another 5-s exposure of the trained odor followed by 5 s of a novel odor to mimic the behavioral test situation under the microscope. Odor-evoked responses of PPL1-α3 DANs increased from the first to the second trial (Figures 4A and S1A), whereas between trial PPL1-γ2α′1 DAN responses did not evidently change (Figures 4C and S1B). However, PPL1-γ2α′1 DANs exhibited a reduced response to the pre-exposed compared to the novel odor in the 5-s test, whereas PPL1-α3 DANs showed no difference between novel and test odor responses (Figures 4B, 4D, S1A, and S1B).

In addition, consistent with a lack of LI (Figure 1H) and pre-exposure memory (Figure 2F), lower 10^{-6} odor concentration did not increase the odor-evoked responses of PPL1-α3 DANs (Figures 4E and S1C). Together, these results suggest that the increased odor-driven activity of the PPL1-α3 DANs, specifically in the 2nd pre-exposure, is crucial for the formation of an aversive pre-exposure memory. The relative change at test of the pre-exposed odor responses of PPL1-γ2α′1 DANs also implies an exposure-dependent plasticity of their activity, perhaps occurring earlier than that of the α3 DANs.

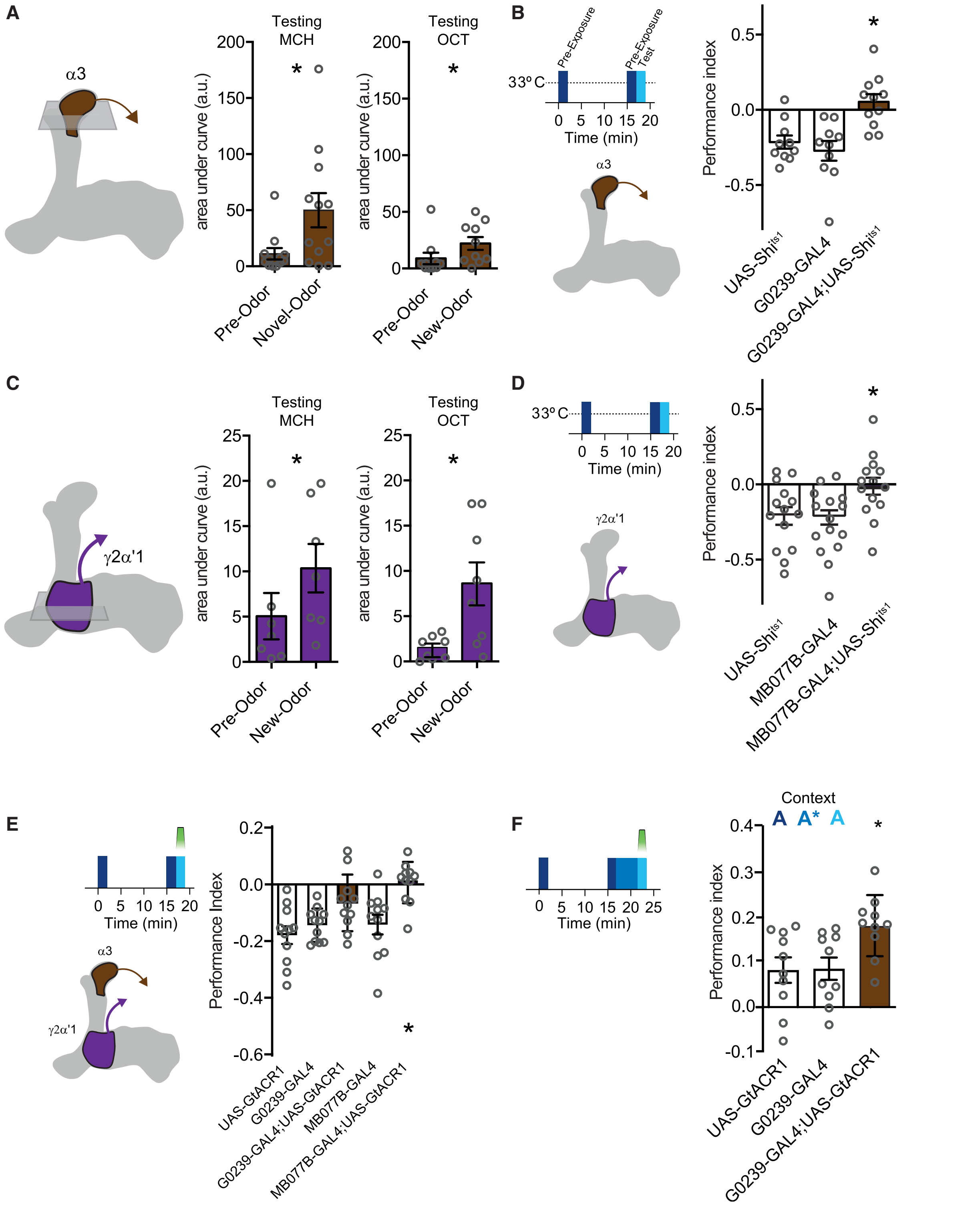

Figure 5. γ2α′1 and α3 MBONs exhibit an odor pre-exposure memory trace and are required for behavioral expression of odor aversion and LI of appetitive memory

(A) Odor pre-exposure reduces the responses of MBON-α3 to that odor compared to a novel odor (for MCH: W(12) = 56, p = 0.0269; for OCT: W(13) = 57, p = 0.0429). Imaging traces are presented in Figure S2.

(B) Blocking output from MBON-α3 impairs pre-exposure memory performance (F[2,28] = 10.50, p = 0.0004, n = 10–11; Tukey's multiple comparisons test: UAS-Shi^{ts1} versus G0239-GAL4, p = 0.7412; UAS-Shi^{ts1} versus G0239-GAL4;UAS-Shi^{ts1}, p = 0.0043; G0239-GAL4 versus G0239-GAL4;UAS-Shi^{ts1}, p = 0.0006).

(legend continued on next page)

Repeated odor presentation depresses odor responses of γ2α′1 and α3 MBONs

The predominant model for *Drosophila* learning is dopamine-driven depression of synapses between odor-specific KCs and MBONs.[29,66–70] We therefore used MBON expression of GCaMP6m to test whether KC-MBON connections underlying the PPL1-α3 and PPL1-γ2α′1 DANs were changed following repeated odor exposure (Figures 5 and S2). Odor responses of MBON-α3 and MBON-γ2α′1 were both reduced compared to their responses to a novel odor (Figures 5A, 5C, and S2). In addition, blocking either the α3 or γ2α′1 MBONs throughout an odor exposure experiment with UAS-*Shi*^{ts1}, or specifically during the test phase with UAS-*GtACR1*, abolished exposure-induced avoidance behavior in the T-maze (Figures 5B, 5D, and 5E). Taken together, these data indicate that spaced odor exposure forms aversive memory that manifests as reduced odor-evoked activity of the α3 and MBON-γ2α′1 MBONs resulting from increased odor-evoked activation of the corresponding DANs.

α3 MBON output is required for expression of latent inhibition of appetitive memory

We last tested whether the activity of the γ2α′1 and α3 MBONs was required for the expression of LI. Flies expressing UAS-*GtACR1* in γ2α′1 and α3 MBONs were subjected to the standard pre-exposure regimen followed by appetitive conditioning (Figure 5F). Blocking output from γ2α′1 MBONs impaired the expression of appetitive memory, so their contribution to LI could not be further tested (data not shown). However, silencing the α3 MBONs during testing by illuminating the flies with green light abolished the effect of LI that was observed in the control flies.

DISCUSSION

In this study, we demonstrate a form of latent inhibition (LI) in *Drosophila* and identify an underlying neuronal mechanism. We find that repeated odor presentation can form a labile self-reinforced aversive memory for that odor, which can temporarily compete with the expression of a newly acquired appetitive memory for that same odor. During memory testing, the conditioned odor should therefore activate both the memory of the pre-exposure (odor-self) and that of the appetitive conditioning (odor-sugar). Importantly, the aversive pre-exposure memory is labile, which means that the LI effect is transient. As a result, the appetitive memory performance exhibits "spontaneous recovery." These results demonstrate that an R model underlies this form of LI in the fly. An A model is not supported because flies acquire an associative reward memory for the odor after pre-exposures of that odor. Instead, the expression of the learned approach performance is impeded by the co-expression of a competing aversive pre-exposure memory. In further support of this R model, the same pre-exposure regimen caused facilitation of a subsequently acquired aversive olfactory memory. In this instance, the pre-exposure memory adds to the new aversive associative memory, rather than competes with an appetitive memory. Last, pre-exposure to the to be non-reinforced odor (the CS−) enhanced subsequent appetitive memory performance but inhibited aversive memory performance—a logical expectation of the CS− acquiring negative valence during pre-exposure.

A defining feature of LI is sensitivity to the consistency of the context in which the pre-exposure, learning, and testing are carried out.[71] Changing between the clear and paper-lined tubes did not impair LI, suggesting that the flies likely consider these to be a similar context. However, if odor pre-exposure, learning, and testing were performed in different contexts (i.e., a copper grid-lined versus a paper-lined or clear tube) LI was abolished. Most strikingly, LI could be restored if copper grid tubes were used to provide the same context during pre-exposure and testing. In line with prior theories and studies of LI in other animals,[72,73] these results suggest that flies learn an association between the odor and the context in which it is experienced during the non-reinforced pre-exposure. As a result, the pre-exposure memory gains context-dependence, and our experiments show it is not retrieved and therefore does not interfere with the newer appetitive memory if the context is different when memory is tested. The failure to retrieve the pre-exposure memory in a different context manifests as a loss of LI—the appetitive memory is fully expressed. Our study therefore reveals that the context-dependency of LI results from the ability (correct context, LI evident) or inability (wrong context, no LI) to retrieve the pre-exposure memory. In addition, context only plays a role in the expression of the pre-exposure memory when it is in conflict with a subsequently acquired appetitive memory. Further work will be required to define what the flies recognize as a "change of context." There are many possibilities including background odors, tube/paper texture, relative luminance, and other flies in the group.

We found that the odor-driven activity of γ2α′1 and α3 DANs increased with repeated odor pre-exposure and that they were required for the formation of the odor pre-exposure memory. In addition, the odor-specific responses of the corresponding

(C) Odor pre-exposure reduces responses of MBON-γ2α′1 to that odor compared to a novel odor (for MCH: W(7) = 24, p = 0.0436; for OCT: t(7) = 2.689, p = 0.0311). Imaging traces are presented in Figure S2.

(D) Blocking output from MBON-γ2α′1 impairs pre-exposure memory performance (F[2,45] = 5.063, p = 0.0104, n = 16; Tukey's multiple comparisons test: UAS-*Shi*^{ts1} versus MB077B-GAL4, p = 0.9823; UAS-*Shi*^{ts1} versus MB077B-GAL4;UAS-*Shi*^{ts1}, p = 0.0284; MB077B-GAL4 versus MB077B-GAL4;UAS-*Shi*^{ts1}, p = 0.0181).

(E) Restricting MBON block to the test phase using GtACR1 reveals a requirement for MBON-γ2α′1 but not MBON-α3 to express the pre-exposure memory (F[4,50] = 7.188, p = 0.0001, n = 11; Šídák's multiple comparisons test: UAS-GtACR1 versus G0239-GAL4, p = 0.9435; UAS-*GtACR1* versus G0239-GAL4;UAS-*GtACR1*, p = 0.0337; G0239-GAL4 versus G0239-GAL4;UAS-*GtACR1*, p = 0.2668; UAS-*GtACR1* versus MB077B-GAL4, p = 0.9249; UAS-*GtACR1* versus MB077B-GAL4;UAS-*GtACR1*, p = 0.0001; MB077B-GAL4 versus MB077B-GAL4;UAS-*GtACR1*, p = 0.0027).

(F) Blocking MBON-α3 during memory testing impairs the expression of LI of appetitive memory. The inhibitory effect of odor X pre-exposure on appetitive memory is not observed when α3 MBONs are blocked at retrieval (F[2,27] = 5.063, p = 0.0136, n = 10; Tukey's multiple comparisons test: UAS-*GtACR1* versus G0239-GAL4, p = 0.9965; UAS-*GtACR1* versus G0239-GAL4;UAS-*GtACR1*, p = 0.0248; G0239-GAL4 versus G0239-GAL4;UAS-*GtACR1*, p = 0.0297). All behavioral pre-exposure experiments were performed in the context A (clear tubes) for pre-exposure and testing phases.

MBONs were depressed following pre-exposure. We therefore conclude that ramping odor-driven DAN activity assigns negative value to the odor itself by depressing odor-specific KC connections onto the γ2α′1 and α3 MBONs. In support of this model, repeated pre-exposure of flies to a lower and less innately aversive odor concentration did not increase the activity of the α3 DANs or form an aversive pre-exposure memory. Importantly, reduced odor activation of the approach-directing γ2α′1 and α3 MBONs is sufficient to account for the aversive nature of pre-exposure memory. Moreover, both expression of pre-exposure memory and LI are abolished if the α3 MBONs are blocked during testing, confirming the model that LI is produced by the expression of the aversive pre-exposure memory competing with that of the associative reward memory believed to be represented as depression of conditioned odor responses of γ5, β′2, and α1 MBONs.[33,68,74]

Several prior *Drosophila* studies have documented changes in odor-driven behavior following different regimens of odor exposure, many of which employed longer durations or more trials than those employed here, and that produced shorter-lived inhibitory effects.[17,18,75] One of these studies described odor-driven activity of the PPL1-α′3 DANs, and subsequent depression of odor-specific responses of the α′3 MBONs to underlie how flies can become familiar with an odor following repeated short exposures.[17] In contrast, we show that two longer and spaced odor exposures produce an aversive memory that manifests as plasticity of γ2α′1 and α3 DANs and MBONs. Moreover, whereas we show a retrieval defect underlies LI of appetitive memory, a reduced attention/familiarity/habituation to the odor following pre-exposures would be expected to result in a subsequent acquisition defect (and A model), likely of both appetitive and aversive learning. It will nevertheless be important to understand how these different types of olfactory experience, and their supporting plasticity mechanisms, are represented and combined in the brain.

LI has often been compared to memory extinction,[76] and our work in the fly shows that very similar neuronal mechanisms and learning models account for both of these phenomena. Pre-exposure learning in *Drosophila* appears to follow similar rules to extinction learning following aversive olfactory conditioning; 2 spaced trials with 15-min ITI are more efficient than massed training with 1-min ITI,[34] and in both cases, a resulting parallel opposing odor-nothing memory inhibits the retrieval/expression of the odor-punishment or odor-reward memory.[34,77] The obvious difference is that the interfering non-reinforced odor memory is formed before learning for LI and after learning for extinction.

Our studies of learning, extinction, and LI suggest that flies acquire and store all of their experience (rewarded and unrewarded, punished and unpunished) as parallel memory traces.[34,35,37,77,78] As a result, when evoked by an appropriate cue, the relevant experiences are compared/combined at the time of retrieval to determine the most fitting behavioral outcome. Such a model is reminiscent of the Miller and Matzel[79] comparator hypothesis, devised mostly from experiments in rodents.[11] Because recent studies suggest similar processes underlie extinction of fear in flies, rodents, and humans,[34,80–84] it seems likely that the form and mechanism of LI we describe here will also be relevant across phyla.

STAR★METHODS

Detailed methods are provided in the online version of this paper and include the following:

- KEY RESOURCES TABLE
- RESOURCE AVAILABILITY
  - Lead contact
  - Materials availability
  - Data and code availability
- EXPERIMENTAL MODEL AND SUBJECT DETAILS
  - Fly *strains*
- METHOD DETAILS
  - Behavioral experiments
  - Two-Photon Calcium Imaging
- QUANTIFICATION AND STATISTICAL ANALYSIS
  - Statistical Analysis

SUPPLEMENTAL INFORMATION

Supplemental information can be found online at https://doi.org/10.1016/j.cub.2021.05.056.

ACKNOWLEDGMENTS

We thank G. Rubin, FlyLight, B. Dickson, and the Bloomington Stock Center for flies. We are grateful to Geraldine Wright and members of the Waddell group for discussion. P.V.-G. is funded by the Sloane Robinson/Clarendon Scholarship given by the University of Oxford Clarendon Fund scheme and Keble College, and by the Consejo Nacional de Ciencia y Tecnología, Mexico (CONACYT). S.W. is funded by a Wellcome Principal Research Fellowship (200846/Z/16/Z) and an ERC Advanced Grant (789274).

AUTHOR CONTRIBUTIONS

Designed Research, P.F.J., Z.O., P.V.-G., J.F., and S.W.; Performed Research, P.F.J., Z.O., P.V.-G., J.F., and S.V.-M.; Analyzed Data, P.F.J., Z.O., J.F., P.V.-G., and S.V.-M.; Resources, S.W.; Writing, S.W., P.F.J., Z.O., and J.F.; Supervision, S.W.; Funding Acquisition, S.W.

DECLARATION OF INTERESTS

S.W. is a member of the advisory board of *Current Biology*.

Received: February 19, 2021
Revised: April 29, 2021
Accepted: May 26, 2021
Published: June 18, 2021

REFERENCES

1. Tolman, E.C., and Honzik, C.H. (1930). Introduction and removal of reward, and maze performance in rats. Univ. Calif. Publ. Psychol. *4*, 257–275.
2. Blodgett, H.C. (1929). The effect of the introduction of reward upon the maze performance of rats. Univ. Calif. Publ. Psychol. *4*, 113–134.
3. Lubow, R.E., and Moore, A.U. (1959). Latent inhibition: the effect of non-reinforced pre-exposure to the conditional stimulus. J. Comp. Physiol. Psychol. *52*, 415–419.
4. Weiss, K.R., and Brown, B.L. (1974). Latent inhibition: a review and a new hypothesis. Acta Neurobiol. Exp. (Warsz.) *34*, 301–316.
5. Mackintosh, N.J. (1975). A theory of attention: variations in the associability of stimuli with reinforcement. Psychol. Rev. *82*, 276–298.

6. Lubow, R.E., Schnur, P., and Rifkin, B. (1976). Latent inhibition and conditioned attention theory. J. Exp. Psychol. Anim. Behav. Process. *2*, 163–174.

7. Wagner, A.R. (1976). Priming in STM: an information-processing mechanism for self-generated or retrieval-generated depression in performance. In Habituation: Perspectives from Child Development, Animal Behavior, and Neurophysiology, T.J. Tighe, and R.N. Leaton, eds. (Erlbaum), pp. 95–128.

8. Miller, R.R., Kasprow, W.J., and Schachtman, T.R. (1986). Retrieval variability: sources and consequences. Am. J. Psychol. *99*, 145–218.

9. Weiner, I. (1990). Neural substrates of latent inhibition: the switching model. Psychol. Bull. *108*, 442–461.

10. Bouton, M.E. (1993). Context, time, and memory retrieval in the interference paradigms of Pavlovian learning. Psychol. Bull. *114*, 80–99.

11. Escobar, M., and Miller, R.R. (2010). Latent inhibition: acquisition or performance deficit? In Latent Inhibition: Cognition, Neuroscience and Applications to Schizophrenia, R.E. Lubow, and I. Weiner, eds. (Cambridge University Press), pp. 62–93.

12. Wagner, A.R. (1981). SOP: a model of automatic memory processing in animal behavior. In Information Processing in Animals: Memory Mechanisms, N.E. Spear, and R.R. Miller, eds. (Erlbaum), pp. 5–47.

13. Wagner, A.R. (2003). Context-sensitive elemental theory. Q. J. Exp. Psychol. B *56*, 7–29.

14. Dudai, Y. (1977). Properties of learning and memory in *Drosophila melanogaster*. J. Comp. Physiol. A Neuroethol. Sens. Neural Behav. Physiol. *114*, 69–89.

15. Tempel, B.L., Bonini, N., Dawson, D.R., and Quinn, W.G. (1983). Reward learning in normal and mutant *Drosophila*. Proc. Natl. Acad. Sci. USA *80*, 1482–1486.

16. Colomb, J., Grillenzoni, N., Stocker, R.F., and Ramaekers, A.R.I. (2007). Complex behavioural changes after odour exposure in *Drosophila* larvae. Anim. Behav. *73*, 587–594.

17. Hattori, D., Aso, Y., Swartz, K.J., Rubin, G.M., Abbott, L.F., and Axel, R. (2017). Representations of novelty and familiarity in a mushroom body compartment. Cell *169*, 956–969.e17.

18. Semelidou, O., Acevedo, S.F., and Skoulakis, E.M. (2018). Temporally specific engagement of distinct neuronal circuits regulating olfactory habituation in *Drosophila*. eLife *7*, 1–25.

19. Chandra, S.B.C., Wright, G.A., and Smith, B.H. (2010). Latent inhibition in the honey bee, Apis mellifera: is it a unitary phenomenon? Anim. Cogn. *13*, 805–815.

20. Fernández, V.M., Giurfa, M., Devaud, J.M., and Farina, W.M. (2012). Latent inhibition in an insect: the role of aminergic signaling. Learn. Mem. *19*, 593–597.

21. Bazhenov, M., Huerta, R., and Smith, B.H. (2013). A computational framework for understanding decision making through integration of basic learning rules. J. Neurosci. *33*, 5686–5697.

22. Beck, C.D.O., Schroeder, B., and Davis, R.L. (2000). Learning performance of normal and mutant Drosophila after repeated conditioning trials with discrete stimuli. J. Neurosci. *20*, 2944–2953.

23. Yu, D., Akalal, D.-B.G., and Davis, R.L. (2006). *Drosophila* α/β mushroom body neurons form a branch-specific, long-term cellular memory trace after spaced olfactory conditioning. Neuron *52*, 845–855.

24. Kim, Y.-C., Lee, H.-G., and Han, K.-A. (2007). D1 dopamine receptor dDA1 is required in the mushroom body neurons for aversive and appetitive learning in *Drosophila*. J. Neurosci. *27*, 7640–7647.

25. Tomchik, S.M., and Davis, R.L. (2009). Dynamics of learning-related cAMP signaling and stimulus integration in the *Drosophila* olfactory pathway. Neuron *64*, 510–521.

26. Qin, H., Cressy, M., Li, W., Coravos, J.S., Izzi, S.A., and Dubnau, J. (2012). Gamma neurons mediate dopaminergic input during aversive olfactory memory formation in *Drosophila*. Curr. Biol. *22*, 608–614.

27. Zhang, S., and Roman, G. (2013). Presynaptic inhibition of gamma lobe neurons is required for olfactory learning in Drosophila. Curr. Biol. *23*, 2519–2527.

28. Boto, T., Louis, T., Jindachomthong, K., Jalink, K., and Tomchik, S.M. (2014). Dopaminergic modulation of cAMP drives nonlinear plasticity across the *Drosophila* mushroom body lobes. Curr. Biol. *24*, 822–831.

29. Hige, T., Aso, Y., Modi, M.N., Rubin, G.M., and Turner, G.C. (2015). Heterosynaptic plasticity underlies aversive olfactory learning in Drosophila. Neuron *88*, 985–998.

30. Owald, D., and Waddell, S. (2015). Olfactory learning skews mushroom body output pathways to steer behavioral choice in Drosophila. Curr. Opin. Neurobiol. *35*, 178–184.

31. Burke, C.J., Huetteroth, W., Owald, D., Perisse, E., Krashes, M.J., Das, G., Gohl, D., Silies, M., Certel, S., and Waddell, S. (2012). Layered reward signalling through octopamine and dopamine in *Drosophila*. Nature *492*, 433–437.

32. Lin, S., Owald, D., Chandra, V., Talbot, C., Huetteroth, W., and Waddell, S. (2014). Neural correlates of water reward in thirsty *Drosophila*. Nat. Neurosci. *17*, 1536–1542.

33. Huetteroth, W., Perisse, E., Lin, S., Klappenbach, M., Burke, C., and Waddell, S. (2015). Sweet taste and nutrient value subdivide rewarding dopaminergic neurons in *Drosophila*. Curr. Biol. *25*, 751–758.

34. Felsenberg, J., Jacob, P.F., Walker, T., Barnstedt, O., Edmondson-Stait, A.J., Pleijzier, M.W., Otto, N., Schlegel, P., Sharifi, N., Perisse, E., et al. (2018). Integration of parallel opposing memories underlies memory extinction. Cell *175*, 709–722.e15.

35. Jacob, P.F., and Waddell, S. (2020). Spaced training forms complementary long-term memories of opposite valence in Drosophila. Neuron *106*, 977–991.e4.

36. McCurdy, L.Y., Sareen, P., Davoudian, P.A., and Nitabach, M.N. (2021). Dopaminergic mechanism underlying reward-encoding of punishment omission during reversal learning in Drosophila. Nat. Commun. *12*, 1115.

37. Das, G., Klappenbach, M., Vrontou, E., Perisse, E., Clark, C.M., Burke, C.J., and Waddell, S. (2014). Drosophila learn opposing components of a compound food stimulus. Curr. Biol. *24*, 1723–1730.

38. Galili, D.S., Dylla, K.V., Lüdke, A., Friedrich, A.B., Yamagata, N., Wong, J.Y.H., Ho, C.H., Szyszka, P., and Tanimoto, H. (2014). Converging circuits mediate temperature and shock aversive olfactory conditioning in Drosophila. Curr. Biol. *24*, 1712–1722.

39. Kirkhart, C., and Scott, K. (2015). Gustatory learning and processing in the *Drosophila* mushroom bodies. J. Neurosci. *35*, 5950–5958.

40. Tomchik, S.M. (2013). Dopaminergic neurons encode a distributed, asymmetric representation of temperature in Drosophila. J. Neurosci. *33*, 2166, 76a.

41. Krashes, M.J., and Waddell, S. (2008). Rapid consolidation to a radish and protein synthesis-dependent long-term memory after single-session appetitive olfactory conditioning in Drosophila. J. Neurosci. *28*, 3103–3113.

42. Domjan, M. (1972). CS preexposure in taste-aversion learning: effects of deprivation and preexposure duration. Learn. Motiv. *3*, 389–402.

43. Clarke, M.E., and Hupka, R.B. (1974). The effects of stimulus duration and frequency of daily preconditioning stimulus exposures on latent inhibition in Pavlovian conditioning of the rabbit nictitating membrane response. Bull. Psychon. Soc. *4*, 225–228.

44. De la Casa, G., and Lubow, R.E. (1995). Latent inhibition in conditioned taste aversion: the roles of stimulus frequency and duration and the amount of fluid ingested during preexposure. Neurobiol. Learn. Mem. *64*, 125–132.

45. Morillas, E., González, F., and Hall, G. (2019). Facilitation and retardation of flavor preference conditioning following prior exposure to the flavor conditioned stimulus. Learn. Behav. *47*, 177–186.

46. Gaztañaga, M., Aranda-Fernández, P.E., Díaz-Cenzano, E., and Chotro, M.G. (2015). Latent inhibition and facilitation of conditioned taste aversion in preweanling rats. Dev. Psychobiol. *57*, 96–104.

47. DasGupta, S., and Waddell, S. (2008). Learned odor discrimination in Drosophila without combinatorial odor maps in the antennal lobe. Curr. Biol. *18*, 1668–1674.

48. Sandoz, J.C., Laloi, D., Odoux, J.F., and Pham-Delègue, M.H. (2000). Olfactory information transfer in the honeybee: compared efficiency of classical conditioning and early exposure. Anim. Behav. *59*, 1025–1034.

49. Locatelli, F.F., Fernandez, P.C., Villareal, F., Muezzinoglu, K., Huerta, R., Galizia, C.G., and Smith, B.H. (2013). Nonassociative plasticity alters competitive interactions among mixture components in early olfactory processing. Eur. J. Neurosci. *37*, 63–79.

50. Dolzer, J., Fischer, K., and Stengl, M. (2003). Adaptation in pheromone-sensitive trichoid sensilla of the hawkmoth Manduca sexta. J. Exp. Biol. *206*, 1575–1588.

51. Zufall, F., and Leinders-Zufall, T. (2000). The cellular and molecular basis of odor adaptation. Chem. Senses *25*, 473–481.

52. Zars, T., Fischer, M., Schulz, R., and Heisenberg, M. (2000). Localization of a short-term memory in Drosophila. Science *288*, 672–675.

53. Dubnau, J., Grady, L., Kitamoto, T., and Tully, T. (2001). Disruption of neurotransmission in Drosophila mushroom body blocks retrieval but not acquisition of memory. Nature *411*, 476–480.

54. McGuire, S.E. (2001). The role of Drosophila mushroom body signaling in olfactory memory. Science *293*, 1330–1333.

55. Krashes, M.J., Keene, A.C., Leung, B., Armstrong, J.D., and Waddell, S. (2007). Sequential use of mushroom body neuron subsets during Drosophila odor memory processing. Neuron *53*, 103–115.

56. Trannoy, S., Redt-Clouet, C., Dura, J.M., and Preat, T. (2011). Parallel processing of appetitive short- and long-term memories in Drosophila. Curr. Biol. *21*, 1647–1653.

57. Kitamoto, T. (2001). Conditional modification of behavior in *Drosophila* by targeted expression of a temperature-sensitive shibire allele in defined neurons. J. Neurobiol. *47*, 81–92.

58. Mohammad, F., Stewart, J.C., Ott, S., Chlebikova, K., Chua, J.Y., Koh, T.W., Ho, J., and Claridge-Chang, A. (2017). Optogenetic inhibition of behavior with anion channelrhodopsins. Nat. Methods *14*, 271–274.

59. Wang, J.W., Wong, A.M., Flores, J., Vosshall, L.B., and Axel, R. (2003). Two-photon calcium imaging reveals an odor-evoked map of activity in the fly brain. Cell *112*, 271–282.

60. Schwaerzel, M., Monastirioti, M., Scholz, H., Friggi-Grelin, F., Birman, S., and Heisenberg, M. (2003). Dopamine and octopamine differentiate between aversive and appetitive olfactory memories in Drosophila. J. Neurosci. *23*, 10495–10502.

61. Claridge-Chang, A., Roorda, R.D., Vrontou, E., Sjulson, L., Li, H., Hirsh, J., and Miesenböck, G. (2009). Writing memories with light-addressable reinforcement circuitry. Cell *139*, 405–415.

62. Aso, Y., Siwanowicz, I., Bräcker, L., Ito, K., Kitamoto, T., and Tanimoto, H. (2010). Specific dopaminergic neurons for the formation of labile aversive memory. Curr. Biol. *20*, 1445–1451.

63. Aso, Y., Herb, A., Ogueta, M., Siwanowicz, I., Templier, T., Friedrich, A.B., Ito, K., Scholz, H., and Tanimoto, H. (2012). Three dopamine pathways induce aversive odor memories with different stability. PLoS Genet. *8*, e1002768.

64. Liu, C., Plaçais, P.Y., Yamagata, N., Pfeiffer, B.D., Aso, Y., Friedrich, A.B., Siwanowicz, I., Rubin, G.M., Preat, T., and Tanimoto, H. (2012). A subset of dopamine neurons signals reward for odour memory in *Drosophila*. Nature *488*, 512–516.

65. Aso, Y., and Rubin, G.M. (2016). Dopaminergic neurons write and update memories with cell-type-specific rules. eLife *5*, 1–15.

66. Séjourné, J., Plaçais, P.-Y., Aso, Y., Siwanowicz, I., Trannoy, S., Thoma, V., Tedjakumala, S.R., Rubin, G.M., Tchénio, P., Ito, K., et al. (2011). Mushroom body efferent neurons responsible for aversive olfactory memory retrieval in *Drosophila*. Nat. Neurosci. *14*, 903–910.

67. Bouzaiane, E., Trannoy, S., Scheunemann, L., Plaçais, P.Y., and Preat, T. (2015). Two independent mushroom body output circuits retrieve the six discrete components of *Drosophila* aversive memory. Cell Rep. *11*, 1280–1292.

68. Owald, D., Felsenberg, J., Talbot, C.B., Das, G., Perisse, E., Huetteroth, W., and Waddell, S. (2015). Activity of defined mushroom body output neurons underlies learned olfactory behavior in Drosophila. Neuron *86*, 417–427.

69. Perisse, E., Owald, D., Barnstedt, O., Talbot, C.B., Huetteroth, W., and Waddell, S. (2016). Aversive learning and appetitive motivation toggle feed-forward inhibition in the Drosophila mushroom body. Neuron *90*, 1086–1099.

70. Handler, A., Graham, T.G.W., Cohn, R., Morantte, I., Siliciano, A.F., Zeng, J., Li, Y., and Ruta, V. (2019). Distinct dopamine receptor pathways underlie the temporal sensitivity of associative learning. Cell *178*, 60–75.e19.

71. De la Casa, L.G., and Pineño, O. (2010). Inter-stage context and time as determinants of latent inhibition. In Latent Inhibition, R.E. Lubow, and I. Weiner, eds. (Cambridge University Press), pp. 40–61.

72. Bouton, M.E., and Swartzentruber, D. (1989). Slow reacquisition following extinction: context, encoding, and retrieval mechanisms. J. Exp. Psychol. Anim. Behav. Process. *15*, 43–53.

73. Westbrook, R.F., Jones, M.L., Bailey, G.K., and Harris, J.A. (2000). Contextual control over conditioned responding in a latent inhibition paradigm. J. Exp. Psychol. Anim. Behav. Process. *26*, 157–173.

74. Yamagata, N., Ichinose, T., Aso, Y., Plaçais, P.Y., Friedrich, A.B., Sima, R.J., Preat, T., Rubin, G.M., and Tanimoto, H. (2015). Distinct dopamine neurons mediate reward signals for short- and long-term memories. Proc. Natl. Acad. Sci. USA *112*, 578–583.

75. Das, S., Sadanandappa, M.K., Dervan, A., Larkin, A., Lee, J.A., Sudhakaran, I.P., Priya, R., Heidari, R., Holohan, E.E., Pimentel, A., et al. (2011). Plasticity of local GABAergic interneurons drives olfactory habituation. Proc. Natl. Acad. Sci. USA *108*, E646–E654.

76. Westbrook, R.F., and Bouton, M.E. (2010). Latent inhibition and extinction: their signature phenomena and the role of prediction error. In Latent Inhibition: Cognition, Neuroscience and Applications to Schizophrenia, R.E. Lubow, and I. Weiner, eds. (Cambridge University Press), pp. 23–39.

77. Felsenberg, J., Barnstedt, O., Cognigni, P., Lin, S., and Waddell, S. (2017). Re-evaluation of learned information in *Drosophila*. Nature *544*, 240–244.

78. Senapati, B., Tsao, C.-H.H., Juan, Y.-A.A., Chiu, T.H., Wu, C.L., Waddell, S., and Lin, S. (2019). A neural mechanism for deprivation state-specific expression of relevant memories in Drosophila. Nat. Neurosci. *22*, 2029–2039.

79. Miller, R.R., and Matzel, L.D. (1988). The comparator hypothesis: a response rule for the expression of associations. In The Psychology of Learning and Motivation: Advances in Research and Theory, G.H. Bower, ed. (Academic Press), pp. 51–92.

80. Luo, R., Uematsu, A., Weitemier, A., Aquili, L., Koivumaa, J., McHugh, T.J., and Johansen, J.P. (2018). A dopaminergic switch for fear to safety transitions. Nat. Commun. *9*, 2483.

81. Salinas-Hernández, X.I., Vogel, P., Betz, S., Kalisch, R., Sigurdsson, T., and Duvarci, S. (2018). Dopamine neurons drive fear extinction learning by signaling the omission of expected aversive outcomes. eLife *7*, 1–25.

82. Kalisch, R., Gerlicher, A.M.V., and Duvarci, S. (2019). A dopaminergic basis for fear extinction. Trends Cogn. Sci. *23*, 274–277.

83. Zhang, X., Kim, J., and Tonegawa, S. (2020). Amygdala reward neurons form and store fear extinction memory. Neuron *105*, 1077–1093.e7.

84. Thiele, M., Yuen, K.S.L., Gerlicher, A.V.M., and Kalisch, R. (2021). A ventral striatal prediction error signal in human fear extinction learning. Neuroimage *229*, 117709.

85. Aso, Y., Hattori, D., Yu, Y., Johnston, R.M., Iyer, N.A., Ngo, T.-T.T.B., Dionne, H., Abbott, L.F.L., Axel, R., Tanimoto, H., and Rubin, G.M. (2014). The neuronal architecture of the mushroom body provides a logic for associative learning. eLife *3*, e04577.

86. Aso, Y., Sitaraman, D., Ichinose, T., Kaun, K.R., Vogt, K., Belliart-Guérin, G., Plaçais, P.-Y.Y., Robie, A.A., Yamagata, N., Schnaitmann, C., et al.

CellPress
OPEN ACCESS

(2014). Mushroom body output neurons encode valence and guide memory-based action selection in Drosophila. eLife *3*, e04580.

87. Pai, T.-P., Chen, C.-C., Lin, H.-H., Chin, A.-L., Lai, J.S.-Y., Lee, P.-T., Tully, T., and Chiang, A.-S. (2013). *Drosophila* ORB protein in two mushroom body output neurons is necessary for long-term memory formation. Proc. Natl. Acad. Sci. USA *110*, 7898–7903.

88. Krashes, M.J., DasGupta, S., Vreede, A., White, B., Armstrong, J.D., and Waddell, S. (2009). A neural circuit mechanism integrating motivational state with memory expression in Drosophila. Cell *139*, 416–427.

89. Klapoetke, N.C., Murata, Y., Kim, S.S., Pulver, S.R., Birdsey-Benson, A., Cho, Y.K., Morimoto, T.K., Chuong, A.S., Carpenter, E.J., Tian, Z., et al. (2014). Independent optical excitation of distinct neural populations. Nat. Methods *11*, 338–346.

90. Chen, T.-W., Wardill, T.J., Sun, Y., Pulver, S.R., Renninger, S.L., Baohan, A., Schreiter, E.R., Kerr, R.A., Orger, M.B., Jayaraman, V., et al. (2013). Ultrasensitive fluorescent proteins for imaging neuronal activity. Nature *499*, 295–300.

91. Schindelin, J., Arganda-Carreras, I., Frise, E., Kaynig, V., Longair, M., Pietzsch, T., Preibisch, S., Rueden, C., Saalfeld, S., Schmid, B., et al. (2012). Fiji: an open-source platform for biological-image analysis. Nat. Methods *9*, 676–682.

92. Pologruto, T.A., Sabatini, B.L., and Svoboda, K. (2003). ScanImage: flexible software for operating laser scanning microscopes. Biomed. Eng. Online *2*, 13.

93. Tully, T., and Quinn, W.G. (1985). Classical conditioning and retention in normal and mutant *Drosophila melanogaster*. J. Comp. Physiol. A Neuroethol. Sens. Neural Behav. Physiol. *157*, 263–277.

94. Shang, Y., Claridge-Chang, A., Sjulson, L., Pypaert, M., and Miesenböck, G. (2007). Excitatory local circuits and their implications for olfactory processing in the fly antennal lobe. Cell *128*, 601–612.

STAR★METHODS

KEY RESOURCES TABLE

REAGENT or RESOURCE	SOURCE	IDENTIFIER
**Experimental models: organisms/strains**		
Drosophila: Canton-S (WT)	Waddell Lab	N/A
Drosophila: MB247-GAL4	Bloomington Drosophila Stock Center;[52]	RRID: BDSC_50742
Drosophila: MB296B-GAL4	Bloomington Drosophila Stock Center;[85,86]	RRID: BDSC_68308
Drosophila: MB058B-GAL4	Bloomington Drosophila Stock Center;[85,86]	RRID: BDSC_68278
Drosophila: MB630B-GAL4	Bloomington Drosophila Stock Center;[85,86]	RRID: BDSC_68334
Drosophila: G0239-Gal4	Bloomington Drosophila Stock Center;[87]	RRID: BDSC_12639
Drosophila: R58E02-GAL4	Bloomington Drosophila Stock Center;[64]	RRID: BDSC_41347
Drosophila: MB504B-GAL4	Bloomington Drosophila Stock Center;[85,86]	RRID: BDSC_68329
Drosophila: MB077B-GAL4	Bloomington Drosophila Stock Center;[85,86]	RRID: BDSC_68316
Drosophila: c061-GAL4;MBGAL80	[88]	N/A
Drosophila: UAS-CsChrimson::mVenus	Bloomington Drosophila Stock Center;[89]	RRID: BDSC_55135
Drosophila: UAS-GCaMP6m	Bloomington Drosophila Stock Center;[90]	RRID: BDSC_42748
Drosophila: UAS-GtACR1	[58]	N/A
Drosophila: UAS-*Shi*^{ts1}	[57]	N/A
**Chemicals, peptides, and recombinant proteins**		
N-Tris	Sigma-Aldrich	Cat#T5691
NaCl	Sigma-Aldrich	Cat#S7653
KCl	Sigma-Aldrich	Cat#P9333
$NaHCO_3$	Sigma-Aldrich	Cat#S6297
NaH_2PO_4	Sigma-Aldrich	Cat#S8282
$CaCl_2$	Sigma-Aldrich	Cat#21115
$MgCl_2$	Sigma-Aldrich	Cat#M1028
Trehalose	Sigma-Aldrich	Cat#T9531
Glucose	Sigma-Aldrich	Cat#G7528
Sucrose	Sigma-Aldrich	Cat# S0389
Mineral Oil	Sigma-Aldrich	Cat#M5904
4-methylcyclohexanol (98%)	Sigma-Aldrich	Cat#153095
3-octanol (99%)	Sigma-Aldrich	Cat#218405
all trans-Retinal	Sigma-Aldrich	Cat#R2500
**Software and algorithms**		
Fiji	NIH;[91]	https://fiji.sc/
MATLAB R2017b	The Mathworks, Natick, MA	https://www.mathworks.com/products/matlab.html
GraphPad Prism 7	GraphPad Software, La Jolla, CA	https://www.graphpad.com/scientific-software/prism/
Adobe Illustrator CC	Adobe Systems, San Jose, CA	https://www.adobe.com/uk/products/illustrator.html
ScanImage 3.8 software	[92]	https://vidriotechnologies.com/

RESOURCE AVAILABILITY

Lead contact

Further information and requests for resources and reagents should be directed to and will be fulfilled by Scott Waddell (scott.waddell@cncb.ox.ac.uk).

Materials availability

All *Drosophila* lines used in this study (see Key resources table) are available from the Bloomington Stock Center, or on request from the Lead Contact.

Data and code availability

The datasets and customized MATLAB and Fiji scripts supporting the current study have not been deposited in a public repository because they are still in development, but are available from the Lead Contact on request and without restriction.

EXPERIMENTAL MODEL AND SUBJECT DETAILS

Fly *strains*

All *Drosophila melanogaster* strains were reared at 25°C and 40%–50% humidity, except where noted, on standard cornmeal-agar food in 12:12 h light:dark cycle. Canton-S flies were used as wild-type (WT). Transgenes were expressed with previously described GAL4 lines: MB247,[52] R58E02-GAL4,[64] MB504B-GAL4, MB296B-GAL4, MB058B-GAL4, MB630B-GAL4 and MB077B-GAL4,[85,86] c061-GAL4;MBGAL80[88] and G0239-GAL4.[87] For behavioral experiments UAS-*Shi*^{ts1} [57]), UAS-CsChrimson::mVenus[89] and UAS-GtACR1[58] were expressed under the control of the respective GAL4–line. For the live-imaging experiments UAS-GCaMP6m[90] was expressed with the respective GAL4. We used mixed sex flies for behavior and imaging experiments.

METHOD DETAILS

Behavioral experiments

Male flies from the GAL4 lines were crossed to UAS-*Shi*^{ts1}, UAS-CsChrimson or UAS-*GtACR1* females, except in the case of the c061-GAL4;MBGAL80 crosses where UAS-*Shi*^{ts1} males were crossed to c061-GAL4;MBGAL80 females. For heterozygous controls GAL4 or UAS-*Shi*^{ts1} flies were crossed to WT. All flies were raised at 25°C and mixed sex populations of 4–8-day-old flies were used in all experiments. Approximately 80-100 flies were placed in a 25 mL vial containing 1% agar (as a water source) and a 20 × 60 mm piece of filter paper for 18–24 h before training and were kept starved for the entire experiment. For experiments to evaluate the requirement for hunger, flies were fed *ad libitum* by housing them in a 25 mL vial containing standard food for 14–22 h before behavioral experiments. Prior to optogenetic experiments all flies were housed on standard cornmeal food supplemented with 1 mM retinal for 3 days, before being starved as described above.

For experiments involving neuronal blockade with UAS-*Shi*^{ts1}, the time courses of the temperature shifting are provided alongside each graph of memory performance. For *Shi*^{ts1} experiments, flies were transferred to the restrictive 32°C 30 min before the targeted time, except where noted, to allow for acclimatization to the new temperature.

All behavioral experiments were performed using a standard T-Maze and 4-methylcyclohexanol (MCH) and 3-octanol (OCT) diluted in mineral oil (MO) were used as the odors.

For repeated odor exposure/odor pre-exposure experiments an odor X (1:10^{-6} or 1:10^{-3} diluted in MO, either MCH or OCT) or mineral oil (MO) was presented twice for 2 min with an inter trial interval (ITI) of 15 min.

When assessing LI, odor pre-exposure was followed by appetitive olfactory conditioning, performed essentially according to Krashes and Waddell:[41] flies were exposed for 2 min to odor Y without reinforcement, in a tube with dry filter paper (the conditioned stimulus -, CS-), 30 s of clean air, then 2 min with odor X with saturated 5.8M sucrose, dried on a filter paper (the conditioned stimulus+, CS+). For assessing facilation of aversive memory, odor pre-exposure was followed by aversive olfactory conditioning:[69,93] flies recieved 1 min with odor X paired with twelve 30 V electric shocks, except where noted, at 5 s intervals (CS+), 45 s of clean air, 1 min with odor Y without reinforcement.

When experiments involved neuronal inhibition using GtACR1, the presentation of odors during the pre-exposure or testing phase was paired with continuous green light (three high-power LEDs [700 mA, centered at 530 nm] were mounted on one arm of the T-maze).

Immediately after the second exposure, except where noted, flies are given the choice (2 min in darkness) between the pre-exposed odor A and an alternative odor B (novel odor). Performance Index was calculated as the number of flies in the odor A arm minus the number in the odor B arm, divided by the total number of flies.[69,93]

When experiments involved neuronal activation using CsChrimson, the presentation of the CS+ odor during the training phase was paired with the presentation of red light (three high-power LEDs [700 mA, centered at 630 nm] were mounted on one arm of the T-maze and triggered for 1 ms at 500 Hz), and the electric shocks were omitted.

Memory performance was assessed by testing flies for their odor-preference between the CS- and the CS+ odors for 2 min in darkness (except where noted). Performance Index was calculated as the number of flies in the CS+ arm minus the number in the CS- arm,

divided by the total number of flies. For all behavioral experiments, a single sample, or n, represents the average performance score from two reciprocally trained groups of flies.

Naive avoidance was performed as essentially as described.[68] An important alteration of the protocol was to use T-maze tubes lined with filter paper and to replace the filter papers and clean the tubes in between experiments, so that odor concentrations did not accumulate in the tubes. This was particularly important to observe approach toward lower 10^{-6} concentrations. Untrained flies were given 2 min in darkness to choose between a diluted odor (OCT or MCH, either $1{:}10^{-6}$ or $1{:}10^{-3}$) and air bubbled through mineral oil in the T-Maze. Performance Index was calculated as the number of flies in the odor arm minus the number in the air arm, divided by the total number of flies.

Two-Photon Calcium Imaging

All flies were raised at 25°C and 3–8 day-old male and female flies were used in all experiments. Imaging experiments were performed essentially as described previously.[34,35,68,69] In brief, flies were immobilized on ice and mounted in a custom-made chamber allowing free movement of the antennae and legs. The head capsule was opened under room temperature carbogenated (95% O2, 5% CO2) buffer solution (103 mM NaCl, 3 mM KCl, 5mM N-Tris, 10 mM trehalose, 10 mM glucose, 7mM sucrose, 26 mM NaHCO3, 1mM NaH2PO4, 1.5 mM CaCl2, 4mM MgCl2, osmolarity 275 mOsm, pH 7.3) and the fly, in the recording chamber, was placed under the Two-Photon microscope (Scientifica).

Flies were exposed to odors under the microscope using essentially the same regimens and odor concentrations as those in the behavioral experiments. Flies were subjected to a constant air stream, carrying vapor from mineral oil solvent (air). For the odor pre-exposures, an odor stream was added to the air for 2 min. Flies in the custom chamber were then removed from the microscope and rested for 15 min until being returned to the microscope and given the 2nd odor exposure. The carbogenated buffer was changed before each re-exposure. To emulate the testing phase, after the 2nd exposure, the flies were sequentially exposed to the re-exposed odor and a novel odor, each for 5 s, interspersed by 30 s of air. As in the behavior experiments the odors were MCH and OCT, and they were used reciprocally. GCaMP responses were measured in the relevant DANs and MBONs during pre-exposure and test phases.

One hemisphere of the brain was randomly selected to image the dendritic field of each MBON and the presynaptic terminals of each DAN. Flies that did not respond to one of the two presented odors were excluded from the analyses in this study. Each n corresponds to a recording from a single fly.

Fluorescence was excited using ~140 fs pulses, 80 MHz repetition rate, centered on 910 nm generated by a Ti-Sapphire laser (Chameleon Ultra II, Coherent). Images of 256 × 256 pixels were acquired at 5.92 Hz, controlled by ScanImage 3.8 software.[92] Odors were delivered using a custom-designed system.[94]

For analysis, two-photon fluorescence images were manually segmented using Fiji.[91] Movement of the animals was small enough such that images did not require registration. For subsequent quantitative analyses, custom Fiji and MATLAB scripts were used. The baseline fluorescence, F_0, was defined for each stimulus response as the mean fluorescence F from 2 s before and up to the point of odor presentation. F/F_0 accordingly describes the fluorescence relative to this baseline. For the imaging data, the area under the curve (AUC) was measured as the integral of F/F_0 during the 5 s odor stimulation.

QUANTIFICATION AND STATISTICAL ANALYSIS

Statistical Analysis

Statistical analyses were performed in GraphPad Prism. All behavioral data were analyzed with an one-sample t test, an unpaired t test or a one-way ANOVA followed by a posthoc Tukey's, Bonferroni's or Šidák's multiple comparisons test. No statistical methods were used to predetermine sample size. For the imaging experiments odor-evoked responses were compared by a paired t test for normally distributed data, otherwise a Wilcoxon matched-pairs signed rank test was used for non-Gaussian distributed data. Normality was tested using the Shapiro-Wilk normality test. For imaging data, a method for outlier identification was run for each dataset (ROUT method), which is based on the False Discovery Rate (FDR). The FDR was set to the highest Q value possible (10%). In datasets in which potential outliers were identified, statistical analyses were performed by removing all odor-evoked responses for those flies. The analyses with or without the outliers were not different, so we decided to maintain and present the complete datasets, which may contain potential outliers.

Current Biology, Volume *31*

Supplemental Information

Prior experience conditionally inhibits

the expression of new learning in *Drosophila*

Pedro F. Jacob, Paola Vargas-Gutierrez, Zeynep Okray, Stefania Vietti-Michelina, Johannes Felsenberg, and Scott Waddell

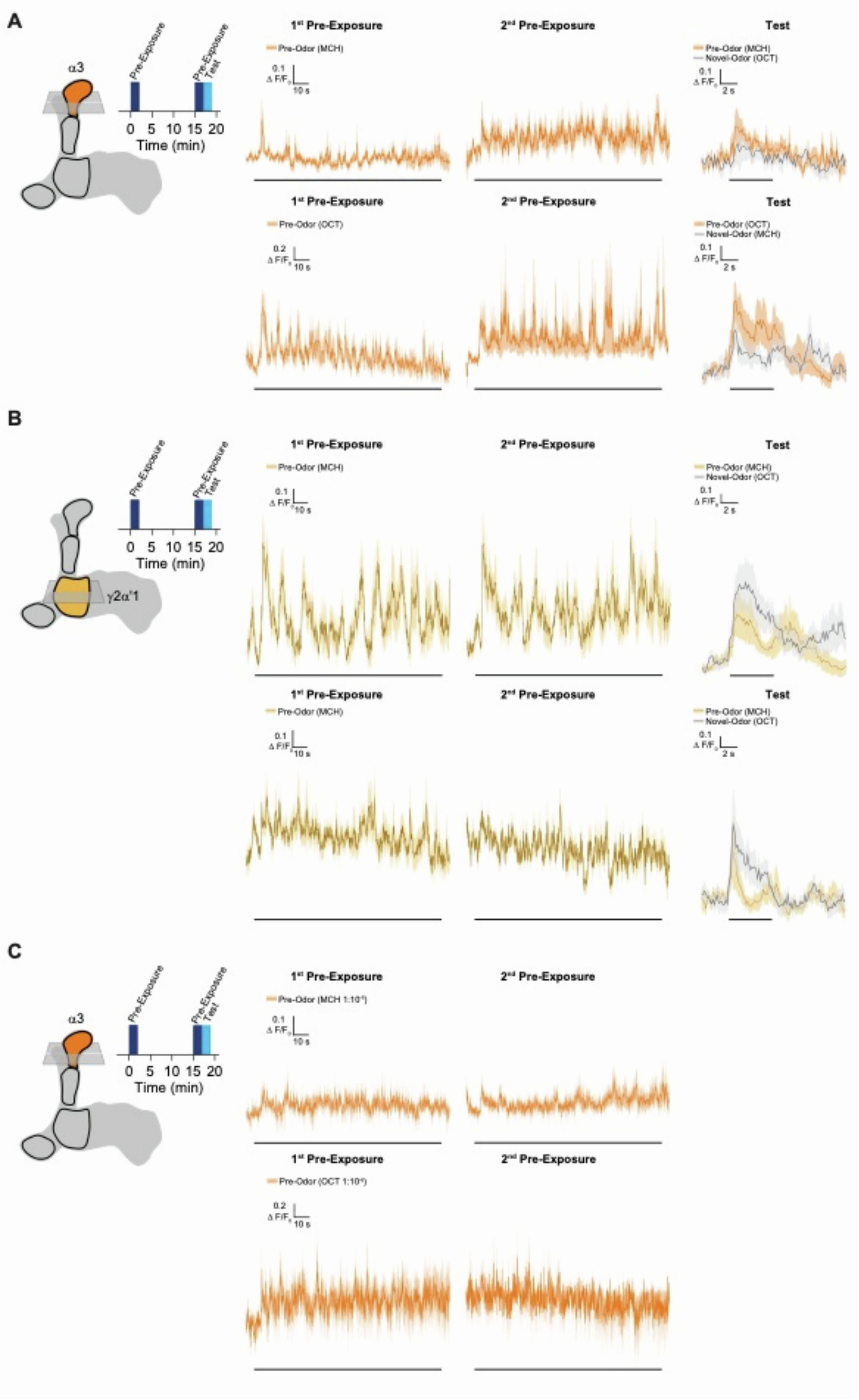

Figure S1. PPL1-α3 and PPL1-γ2α′1 DAN activity is altered following odor exposure. Related to Figure 4.

(**A**) Left: Imaging plane through the presynaptic field of an α3 DAN and training and imaging protocol. Middle: α3 DAN odor-evoked calcium responses during the 1st and 2nd pre-exposures to odor X (Pre-odor; either MCH or OCT, orange). Right: α3 DAN odor-evoked calcium

responses during the testing phase to odor X (Pre-odor, orange) and to odor Y (Novel-odor, gray). (**B**) Left: Imaging plane through the presynaptic field of a γ2α′1 DAN and training and imaging protocol. Middle: γ2α′1 DAN odor-evoked calcium responses during the 1st and 2nd pre-exposures to odor X (Pre-odor; either MCH or OCT, gold). Right: γ2α′1 DAN odor-evoked calcium responses during the testing phase to odor X (Pre-odor, gold) and to odor Y (Novel-odor, gray). (**C**) Left: Imaging plane through the presynaptic field of an α3 DAN and training and imaging protocol. Middle: α3 DAN odor-evoked calcium responses during 1st and 2nd pre-exposures to lower 10^{-6} concentration of odor X (Pre-odor; either 1:10^{-6} MCH or OCT, orange). In all panels, the horizontal line underneath the traces denotes the period of odor presentation. Odor-evoked activity traces show mean (solid line) with SEM (shadow). Data quantification in Figure 4.

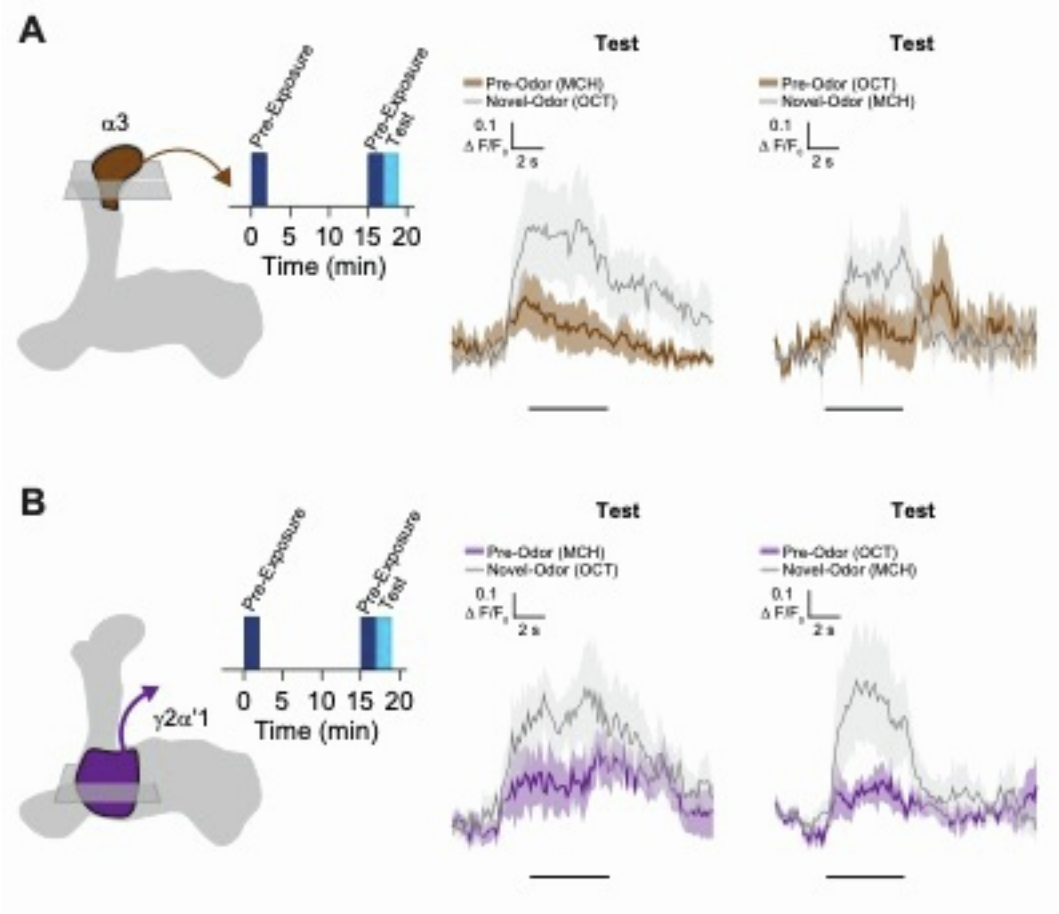

Figure S2. α3 and γ2α′1 MBONs exhibit an odor pre-exposure memory trace. Related to Figure 5.

(**A**) Left: Imaging plane through the dendritic field of an α3 MBON and training and imaging protocol. Right: α3 MBON odor-evoked calcium responses during the testing phase to odor X (Pre-odor, brown) and to odor Y (Novel-odor, gray). (**B**) Left: Imaging plane through the dendritic field of a γ2α′1 MBON and training and imaging protocol. Right: γ2α′1 MBON odor-evoked calcium responses during the testing phase to odor X (Pre-odor, purple) and to the odor Y (Novel-odor, gray). In all panels, the horizontal line underneath the traces denotes the period of odor presentation. Odor-evoked activity traces show mean (solid line) with SEM (shadow). Data quantification in Figure 5.
